# In vivo ligamentogenesis in embroidered poly(lactic-*co*-ε-caprolactone) / polylactic acid scaffolds functionalized by fluorination and hexamethylene diisocyanate cross-linked collagen foams

**DOI:** 10.1007/s00418-022-02156-3

**Published:** 2022-10-29

**Authors:** Maria Kokozidou, Clemens Gögele, Felix Pirrung, Niels Hammer, Christian Werner, Benjamin Kohl, Judith Hahn, Annette Breier, Michaela Schröpfer, Michael Meyer, Gundula Schulze-Tanzil

**Affiliations:** 1 Institute of Anatomy and Cell Biology, Paracelsus Medical University, Nuremberg and Salzburg, Prof. Ernst Nathan Str. 1, 90419 Nuremberg, Germany; 2 Department of Biosciences and Medical Biology, Paris Lodron University Salzburg, Hellbrunnerstraße 34, 5020 Salzburg, Austria; 3 Division of Macroscopic and Clinical Anatomy, Gottfried Schatz Research Center, Medical University of Graz, Harrachgasse 21, 8010 Graz, Austria; 4 Department of Orthopedic and Trauma Surgery, University of Leipzig, Leipzig, Germany; 5 Fraunhofer Institute for Machine Tools and Forming Technology IWU, Nöthnitzer Straße 44, 01187 Dresden, Germany; 6 Department of Traumatology and Reconstructive Surgery, Charité –Universitätsmedizin Berlin, corporate member of Freie Universität Berlin, Humboldt-Universität Zu Berlin, Campus Benjamin Franklin, Hindenburgdamm 30, 12203 Berlin, Germany; 7 Workgroup Bio-Engineering, Department Materials Engineering, Leibniz-Institut für Polymerforschung Dresden e. V. (IPF), Institute Polymers Materials, Hohe Straße 6, 01069 Dresden, Germany; 8 FILK Freiberg Institute gGmbH (FILK), Meißner Ring 1-5, 09599 Freiberg, Germany

**Keywords:** Tissue engineering, Anterior cruciate ligament, Ligamentogenesis, Embroidered, Poly(L-lactide-*co*-ε-caprolactone) (P(LA-CL)), Polylactic acid (PLA) scaffolds, Dynamic nude mice xenograft model

## Abstract

Although autografts represent the gold standard for anterior cruciate ligament (ACL) reconstruction, tissue-engineered ACLs provide a prospect to minimize donor site morbidity and limited graft availability. This study characterizes the ligamentogenesis in embroidered poly(L-lactide-*co*-ε-caprolactone) (P(LA-CL)) / polylactic acid (PLA) constructs using a dynamic nude mice xenograft model. (P(LA-CL))/PLA scaffolds remained either untreated (co) or were functionalized by gas fluorination (F), collagen foam cross-linked with hexamethylene diisocyanate (HMDI) (coll), or F combined with the foam (F + coll). Cell-free constructs or those seeded for 1 week with lapine ACL ligamentocytes were implanted into nude mice for 12 weeks. Following explantation, cell vitality and content, histo(patho)logy of scaffolds (including organs: liver, kidney, spleen), sulphated glycosaminoglycan (sGAG) contents and biomechanical properties were assessed.

Scaffolds did not affect mice weight development and organs, indicating no organ toxicity. Moreover, scaffolds maintained their size and shape and reflected a high cell viability prior to and following implantation. Coll or F + coll scaffolds seeded with cells yielded superior macroscopic properties compared to the controls. Mild signs of inflammation (foreign-body giant cells and hyperemia) were limited to scaffolds without collagen. Microscopical score values and sGAG content did not differ significantly. Although remaining stable after explantation, elastic modulus, maximum force, tensile strength and strain at F_max_ were significantly lower in explanted scaffolds compared to those before implantation, with no significant differences between scaffold subtypes, except for a higher maximum force in F + coll compared with F samples (in vivo). Scaffold functionalization with fluorinated collagen foam provides a promising approach for ACL tissue engineering.

**Graphical abstract:**

**a** Lapine anterior cruciate ligament (LACL): red arrow, posterior cruciate ligament: yellow arrow. Medial anterior meniscotibial ligament: black arrow. **b** Explant culture to isolate LACL fibroblasts. **c** Scaffold variants: co: controls; F: functionalization by gas-phase fluorination; coll: collagen foam cross-linked with hexamethylene diisocyanate (HMDI). **c1-2** Embroidery pattern of the scaffolds. **d** Scaffolds were seeded with LACL fibroblasts using a dynamical culturing approach as depicted. **e** Scaffolds were implanted subnuchally into nude mice, fixed at the nuchal ligament and sacrospinal muscle tendons. **f** Two weeks after implantation. **g** Summary of analyses performed. Scale bars 1 cm (**b**,** d**), 0.5 cm (**c**). (sketches drawn by G.S.-T. using Krita 4.1.7 [Krita foundation, The Netherlands]).

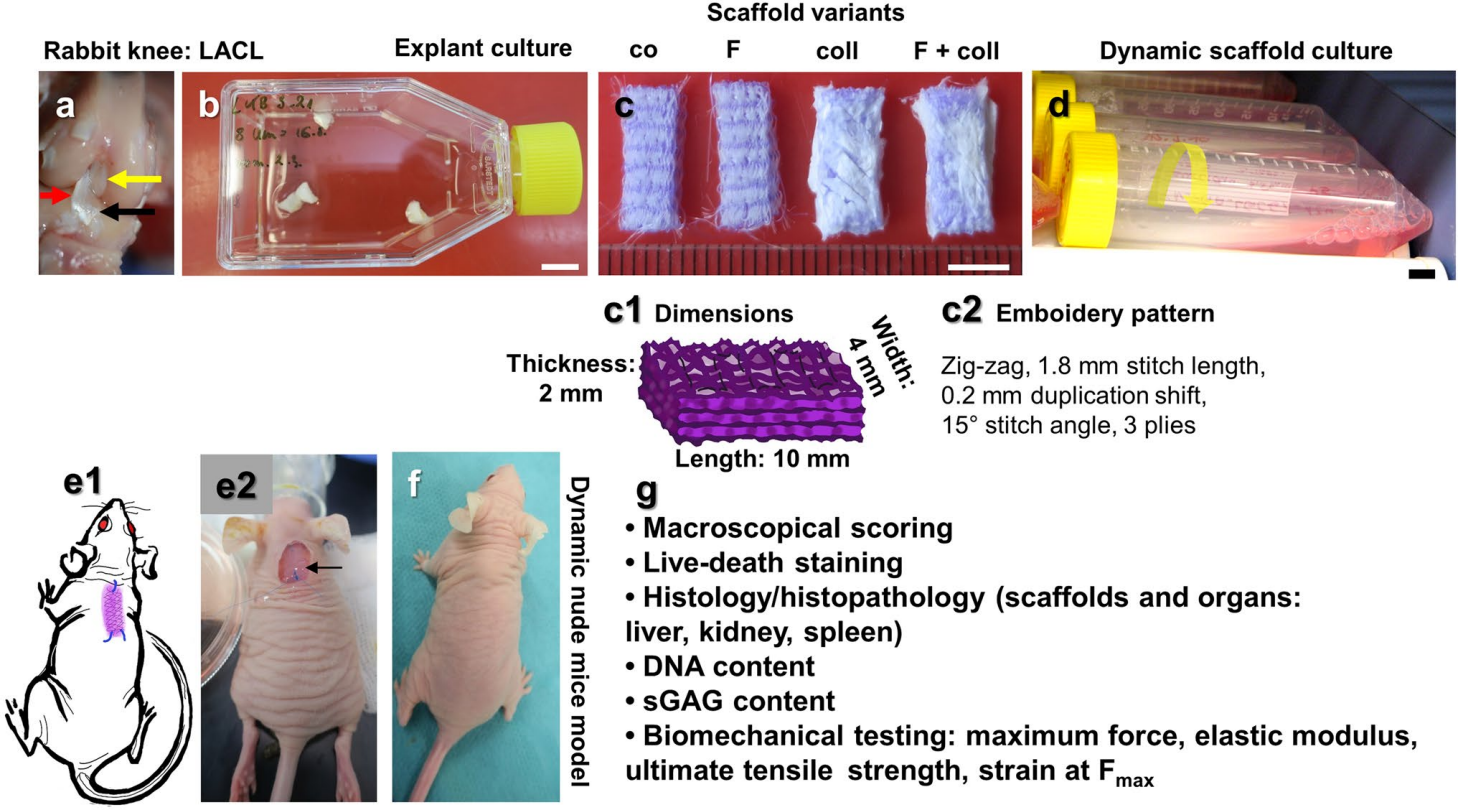

**Supplementary Information:**

The online version contains supplementary material available at 10.1007/s00418-022-02156-3.

## Introduction

The anterior cruciate ligament (ACL) helps to maintain knee joint stability and is one of the most commonly injured ligaments. Ruptured ACLs are usually reconstructed by autografts such as hamstring tendons. To avoid donor site morbidity and limited autograft availability, tissue engineered ACLs may present a prospect strategy for the surgical reconstruction after ACL injury (Rathbone et al. 2012; Riley et al. 2018). However, graft materials used as carriers need to demonstrate a high resilience to sustain continuous mechanical loading, degrade slowly, and should not be releasing harmful degradation or wear of friction products. Braided scaffolds based on synthetic polymers or silk showed substantial mechanocompetence, which was promising for ligament tissue engineering (Cédric Laurent 2018). As an alternative scaffold fabrication method to braiding, the embroidery technology allows the generation of scaffolds with a tailored three-dimensional (3D) structure, porosity, and appropriate biomechanical properties, and uses synthetic thread materials (Hahn et al. 2019). The embroidery pattern can be adapted by varying stitch length and angle as well as the duplication shift. In addition, multilayered and multiphase structures can be created. To allow cell adherence after seeding or cell ingrowth, surface characteristics (composition, topology) are of high importance. Unfortunately, most synthetic polymers show poor cell adherence (Hoyer et al. 2014). Therefore, appropriate functionalization strategies are required to increase cell adherence and spreading (Hoyer et al. 2014). Collagen supplementation is a common strategy for scaffold functionalization (Chang et al. 2020; Dong et al. 2016) and was also applied in the present study. Another functionalization opportunity seems to be gas-phase fluorination. Recently, gas-phase fluorination has been demonstrated as a new and effective method of facilitation of fibroblast adhesion (Schröpfer et al. 2020). However, so far, it has not been investigated whether this strategy also affects in vivo biocompatibility. The cell response mechanism to surface modification by fluorination is still unclear (Schröpfer et al. 2020). To assess the main aspects of biocompatibility of tissue-engineered constructs and monitor their effect on ligamentogenesis, the nude mice xenograft model can be used (Dai et al. 2015; Han et al. 2017; Lohan et al. 2018; Ni et al. 2013; Noack et al. 2014; Xu et al. 2014). To study the contribution of cells to ligamentogenesis, we decided to preseed the scaffolds with ligamentocytes. Another approach would be to use mesenchymal stem cells or progenitor cells (Archer et al. 2018) as a more accessible and more abundant cell source for therapeutic approaches compared to ACL ligamentocytes. The attachment of the biomaterial to the autochthonous back muscle system of the mice was expected to provide some additional biomechanical tensional stimuli during mouse movements in addition to those of the shear forces of the skin covering the construct after subcutaneous implantation. This less common approach of a dynamic nude mice xenograft model has been reported previously (Ni et al. 2013; Xu et al. 2014).

In the present study, we investigated a slowly degrading combination of synthetic polymers (poly(lactic-*co*-ε-caprolactone)/polylactic acid)(P(LA-CL)/PLA) functionalized with gas-phase fluorination (F) and collagen foam cross-linked with hexamethylene diisocyanate (HMDI) (coll) for the first time in vivo using the dynamic nude mice xenograft model to assess ligamentogenesis. The combination of P(LA-CL) with PLA allowed lapine ligamentocyte adherence and a biomechanical competence of the construct comparable to the native lapine ACL (LACL) (Hahn et al. 2019). The functionalization of the material combination with gas-phase fluorination and collagen provided promising properties in several in vitro studies (Gögele et al. 2020a, 2021; Hahn et al. 2019).

## Materials and methods

### Preparation and functionalization of scaffolds

A monofilament P(LA-CL) thread (USP 7–0, Gunze Ldt., Osaka, Japan) and a melt spun PLA multifilament consisting of six single filaments (Tt = 155 dtex, Ingeo biopolymer 6202D, NatureWorks, Minnetonka, MN, USA, fiber melt spinning at the Leibniz-Institut für Polymerforschung Dresden e.V. (IPF) (Dresden, Germany)) were used for scaffold fabrication with an embroidery machine (Type TLMX-901, Tajima Industries, Nagoya, Japan). P(LA-CL) served as upper and PLA as lower thread during the embroidery process. Three plies having a zig-zag pattern design (1.8 mm stitch length, 15° stitch angle, and 0.2 mm duplication shift) were stacked and pinned together. A water-soluble non-woven made of polyvinyl alcohol (PVA, Freudenberg Einlagestoffe KG, Weinheim, Germany) was used as an embroidery base material, later washed out by rinsing three times for 30 min in pyrogen-reduced water on a compact shaker (KS 15 A, Edmund Bühler GmbH, Bodelshausen, Germany). After that, the remaining porous scaffolds were dried at room temperature (RT). The porosity of the scaffolds measured previously by µCT (Breier 2015) ranged between 65 and 75% (continuously through the whole structure).

Gas-phase fluorination was performed at the FILK Freiberg Institute (Freiberg, Germany) in a fluorination batch reactor (Fluor-Technik-System GmbH, Lauterbach, Germany), using a mixture of 10% fluorine gas in synthetic air for 60 s. After the fluorination process, the scaffolds selected for additional collagen functionalization were flushed with synthetic air prior to immersion in solved bovine acid collagen and subsequently refibrillated with phosphate buffer and NaCl. The resulting collagen hydrogel pervading the embroidered scaffold was desalted and lyophilized to form a porous foam between the threads. The collagen cross-linking was executed with HMDI in gas phase in a desiccator.

### Scanning electron microscopy

The basis scaffold (co, without gas-phase fluorination) was visualized using scanning electron microscopy (SEM, Philips, XL30 ESEM-FEG, 3 kV, high vacuum, working distance 10 mm). Samples were put on a carbon pad specimen holder and sputtered with gold 10–20 nm.

### LACL isolation

LACL-derived ligamentocytes were harvested from six adult males, healthy 12-month-old ACLs of New Zealand Rabbits, obtained from the regional slaughterhouse. The explanted LACLs were split into 2-mm^2^ pieces and were cultivated in T25 culture flasks in growth medium (Dulbecco's modified Eagle’s medium [DMEM]/Ham’s F12 medium [1:1, Merck KGaA, Darmstadt, Germany] supplemented with 10% fetal bovine serum [FBS, Merck KGaA], 1% penicillin/streptomycin solution [Merck KGaA], 25 μg/ml ascorbic acid [Sigma-Aldrich, Munich, Germany], 2.5 μg/ml amphotericin B [Merck KGaA], MEM amino acid solution [Sigma-Aldrich]). After 7–10 days, ligamentocytes emigrated and were expanded using 0.05% trypsin/0.02% ethylenediaminetetraacetic acid (EDTA) for the subsequent experiments.

### Dynamic ligamentocyte scaffold culture

Four scaffold variants (co, control: unfunctionalized [1], functionalized only by gas-phase fluorination [2] or only HMDI cross-linked collagen foam (coll) [3] or combination of gas-phase fluorination and HMDI cross-linked collagen foam (F + coll) [4], were disinfected in 70% ethanol (ETOH, Applichem GmbH, Darmstadt, Germany) for at least 30 min before being thoroughly rinsed with sterile and hypotonic water with low pyrogen content (Carl Roth GmbH and Ko.KG, Karlsruhe, Germany). The disinfected scaffolds were incubated for 1 h in FBS for protein absorption. Subsequently, the scaffolds were seeded with 8333 ligamentocytes/mm^3^ scaffold, suspended in 5 ml growth medium in TubeSpin bioreactor tubes (TPP, Trasadingen, Switzerland) using a rotator device (Bartelt GmbH, Graz, Austria) with 36 rpm at 37 °C. Culture medium was changed two times a week and cultivation was ceased at day 7–8.

### Dynamic nude mice xenograft model

Female 6–8 weeks old athymic nude mice (NU-Foxn1nu/nu, Charles River Laboratories, Sulzfeld, Germany,* n* = 80) were purchased and held in a 12-h light/12-h dark cycle at 23 °C. Animal experimental protocols were approved by the local animal review board (RUF-55.2.2–2532.2–1011-30), as well as the universities’ internal scientific committee. All experiments were performed in compliance with the animal protection FELASA guidelines. The animals were randomly divided into eight different groups for the four different scaffold types used either seeded or not with LACL ligamentocytes (Table 1) and were weekly weighted as part of the weekly clinical surveillance to observe any influence of the construct on the growth curves of the animals after construct implantation. Prior to implantation, the scaffolds were dynamically cultivated for 7–8 days with LACL ligamentocytes or maintained in growth medium without cells for similar durations, all in the same temperature conditions. Immediately afterwards, implantation into the subnuchal subcutaneous space of the mice followed for 3 months.

**Table 1:** Experimental groups

Groups	Without cells			With cells		
	Explanted		Before I	Explanted		Before I
Analysis after 12 weeks in vivo	Hist., vitality	BA, assays	BA, vitality	Hist., vitality	BA, assays	BA, vitality
co: Scaffolds without functionalization	8–10	6–8	3	10	6–8	3
F: Scaffolds with gas-phase fluorination	8–10	6–8	3	10	6–8	3
coll: Scaffolds with cross-linked collagen foam	8–10	6–8	3	10	6–8	3
F + coll: Scaffolds with gas-phase fluorination and collagen	8–10	6–8	3	10	6–8	3

*Hist* histology, *BA* biomechanical analysis, *I* implantation

The mice received pre-operatively (2 h before surgery) 4 mg/kg body weight Rimadyl (Pfizer, Karlsruhe, Germany) subcutaneously (s.c.), administered as analgesic premedication. Inhalation anesthesia followed by 3% isoflurane (Forene 100%, Abbott, Wiesbaden, Germany) for induction and 1.5% for preservation in oxygen (1.5 L/minute), delivered via an anesthetic mask. For 3 days post-operatively, they received again 4 mg/kg body weight Rimadyl s.c., twice daily every 12 h. For surgery, the subnuchal skin of the back was incised along the spine (0.5 cm) under sterile conditions, a pocket was stumpy prepared beneath the incision and the construct was inserted. Scaffolds were fixed with prolene 6:0 (Ethicon, Bridgewater, NJ, USA) at the nuchal ligament and sacrospinal muscle tendons. The incision was sutured with 6–0 Vicryl (Ethicon, Raritan, NJ, USA). The cell-free scaffolds were treated in the same manner. Twelve weeks after implantation of the constructs, the animals were  sacrificed using CO_2_. The explanted constructs were photo-documented (Canon G9 X, Canon, Öta, Tokyo, Japan), macroscopically scored using a self-designed scoring system (Table 2) according to Lohan et al., (Lohan et al. 2018) and then measured. Internal organs (liver, kidneys, and spleen) were explanted for histopathology.

**Table 2:** Macroscopic score system for ligament explants

Macroscopic score	Points
Color	
White, glossy	2
Rose, rough	1
Other color	0
Surface structure	
Smooth, tight, intact	2
Rough	1
Surface with clefts and holes	0
Shape	
Similar as before implantation	2
Unregular, tight	1
Diffuse, smooth	0
Size	
Similar like before implantation	2
Shrunken or swollen	1
Nearly disappeared	0
Maximum count	8

According to Lohan et al. (2018)

### Viability testing

The scaffolds (in vitro cultured or explanted from the nude mice 12 weeks postoperatively) were incubated in 5 µl/ml fluorescein diacetate (FDA, Sigma-Aldrich, 3 mg/ml dissolved in acetone as a stock solution) and 1 µl/ml propidium iodide (PI, 1% solution, Carl Roth GmbH and Ko.KG) in phosphate-buffered saline (PBS) for 1 min at RT. Viable cells were green and dead cells were red as visualized by confocal laser scanning microscopy (CLSM, SPE-II, Leica Microsystems GmbH, Wetzlar, Germany).

### Histological staining

Selected scaffolds and organs (liver, kidney, and spleen) were fixed overnight in 4% paraformaldehyde solution in PBS (PFA/PBS, Santa Cruz Biotechnology Inc., Dallas, TX, USA). In addition, scaffolds explanted for biomechanical analyses were equilibrated at 4 °C (30 min) before frozen and stored at − 80 °C. The left over pieces after preparing the scaffolds for biomechanical testing were also fixed in 4% PFA/PBS for histopathology. Subsequently, samples were dehydrated using an increasing ethanol series (70, 80, 96 and 99.6%) with each step lasting 4 min followed by 10 min’ xylene (all EtOH and xylene from Carl Roth GmbH and Ko.KG) and then, embedded in paraffin (Shandon Pathcenter, Thermo Scientific, Waltham, MA, USA). Paraffin sections (7 µm thick) were prepared, histologically stained, and analyzed using histological scoring systems adapted from Lohan et al., (Lohan et al. 2018) (Table 3).

**Table 3:** Histological scoring of ligament constructs

Criterion	Observation (points)
Inflammation	No (2), only focally (1), generalized or focally pronounced (0)
Vascularity	Low (2), no/slightly increased (1), severely hyperemic (0)
Capsule	No (2), thin (1), thick (0)
Cellularity	Like ligament (2), increased or diminished in comparison to native ligament (0–1)
Cell distribution	Homogenous (2), inhomogeneous (1–0)
Cell morphology	Mainly elongated (ligamentocyte-like) (2),
	Many round cells (1),
	Mainly inflammatory cells (0), many debris (0)
Orientation	A trend of uniaxial order (ligament-like) (1)
	No major order (0),
Cell-to-ECM ratio	Few cells, many ECM (2),
	Many cells, many ECM (immature tissue) (1),
	Many cells, few ECM or several cell free areas (0.5),
	No ECM (0)
ECM	Dense (2), only moderately dense (1), loose (0)
Fatty tissue	No (2), yes (0–1)
Maximum count	19 points

Adapted from Lohan et al. (2018)

### Hematoxylin–Eosin (H&E)

Paraffin sections (thickness: 7 µm) were deparaffinized using xylene and a series of decreasing aqueous ethanol concentrations (99.6, 96, 80, and 70%) for all histological staining procedures. HE staining was used to get an overview of histoarchitecture of the neotissues. Deparaffinized sections were incubated for 4 min in Harris hematoxylin solution (Sigma-Aldrich), then rinsed in tap water and counterstained for 4 min in eosin (Carl Roth GmbH and Ko.KG). After rinsing with distilled (dist.) water, the sections were dehydrated in a series of ascending alcohol concentrations (70, 80, 96, and 99.6%). Finally, the stained sections were mounted with Entellan (Merck KGaA, Darmstadt, Germany) and analyzed using a light microscope (DM1000 LED, Leica Microsystems GmbH).

### Alcian blue (AB) staining

Alcian blue (AB) staining indicates the deposition of sulphated glycosaminoglycans by a blue color. For AB staining, the deparaffinized sections were incubated for 3 min in 1% acetic acid (Carl Roth GmbH and Ko.KG) and then immersed for 30 min in 1% AB in acetic acid (pH 2.5) (Carl Roth GmbH and Ko.KG). Subsequently, they were rinsed in 3% acetic acid. Cell nuclei were counterstained using nuclear fast red aluminum sulphate solution (Carl Roth GmbH and Ko.KG) for 5 min. The sections were dehydrated in a series of ascending alcohol concentrations (70, 80, 96, and 99.6%) before being embedded in Entellan (Merck KGaA) and kept in the dark until analyzed by light microscopy (DM1000 LED, Leica Microsystems GmbH). AB scoring was performed according to the following criteria:

The staining intensity was slightly or substantially lower or higher as the native rabbit ACL (1 or 0 points) or the staining intensity was similar to the native rabbit ACL (2 points). The staining was homogeneously distributed (2 points) or slightly (1 point) or severely inhomogeneously stained (0 point). Hence, the possible maximum was 4 points.

### Sirius red (SR) staining

SR staining was performed to depict distribution of collagen. Deparaffinized sections were washed in nondistilled (nondist.) water for 4 min. Cell nuclei were stained with Weigerts hematoxylin (MORPHISTO GmbH, Frankfurt am Main, Germany) for 8 min. Afterwards, they were washed with distilled water for 5 s, followed by rinsing in nondistilled water for 10 min and in distilled water for 1 min. The sections were stained using SR (MORPHISTO GmbH) for 60 min, before being twice incubated in 30% acetic acid (Carl Roth GmbH and Ko.KG) for 1 min each and then twice in 96% EtOH for 4 min. Finally, stained sections were mounted with Entellan (Merck KGaA) and analyzed using a light microscope (DM1000 LED, Leica Microsystems GmbH). SR scoring was performed according to the following criteria: The staining intensity was classified as absent (0 point), faint (1 point), clearly detectable (2 points) or intense (3 points). The distribution was described as severely inhomogeneous (0 point), slightly inhomogeneous (1 point), or homogeneously distributed (2 points). Hence, the maximum of available points was 5 points.

### Measurement of total DNA and glycosaminoglycan content

The DNA contents of the scaffolds were measured using CyQuant assay (Invitrogen, Carlsbad, CA, USA) according to the user manual including calf thymus DNA as a standard. Each scaffold was homogenized after biomechanical analysis with a 7-mm stainless-steel bead (RNase and DNase free, sterile, Qiagen, Hilden, Germany) by using TissueLyser LT (Qiagen, Hilden, Germany, 50 Hz, 5 min, RT). The samples were digested using a proteinase K solution (0.5 mg/ml, Carl Roth GmbH and Ko.KG) dissolved in 50 mM Tris/HCl, 1 mM EDTA, 0.5% Tween20, pH 8.5) for 16 h at 56 °C under continuous shaking (36 rpm), before being centrifuged for 30 min at 10,000 rpm. The supernatants were frozen at − 20 °C for 30 min to stop the reaction. A 10-µl sample and 150-µl TE buffer were mixed. Triplicates of specimens (each 25 µl) were transferred to a black 96-well plate with a flat bottom (Brand GmbH, Wertheim, Germany) and 25 µl dye solution (1 × HBSS + dye solution 1:250) was added. Plates were protected from light and incubated at 37 °C for 60 min. The fluorescence was measured at λ = 485 excitation/ *λ* = 530 emission using a fluorometric plate reader (Infinite M200, Tecan Austria GmbH, Grödig, Austria). The cell content of the scaffolds was calculated based on the assumption that each cell contains approximately 7.7 pg DNA (Kim et al. 1988).

The lysates were diluted in phosphate-buffered EDTA (PBE) buffer (100 mM Na_2_HPO_4_ and 5 mM EDTA, pH 8.0) to quantify the sulphated glycosaminoglycan (sGAG) contents, before the dimethylmethylene blue (DMMB, AppliChem, Darmstadt, Germany) dyeing solution (8.9 mM DMMB hydrochloride in 600 mg glycerine, 467 mg NaCl and 200 ml of distilled water) was added. The absorption shifts from *λ* = 525 nm to *λ* = 595 nm were measured. sGAG content was calculated based on chondroitin sulphate sodium salt from shark cartilage (Sigma-Aldrich) as a standard (Infinite M200, Tecan Austria GmbH). The sGAG content was divided through the calculated cell number.

### Biomechanical analyses

Biomechanical properties of the scaffolds cultured for only 1 week in vitro before implantation and those explanted after 3-month incubation in vivo were measured. Biomechanical analysis included maximum force (F_max_), elastic modulus, tensile strength and strain at F_max_. Prior to the mechanical tests, the samples were stored at − 80 °C. The time between specimen removal from the freezer and testing was kept to a minimum. Prior to the testing, the samples were thawed quickly to reach room temperature (20 °C). A custom 3D-printed punch was used to stamp a dog-bone shape from the specimens (Nelson et al. 2020). This dog-bone shape shows a slender midsection and wider ends, resulting in a reduced cross-sectional area and therefore standardized failure sites at the samples’ midsections. Removed tissues following the tapering were kept in 4% PFA for further analyses (histopathology). To determine the CSA in the failure zone, the samples’ midsection was carefully molded in silicone (Panasil initial contact; Kettenbach, Eschenburg, Germany). The silicone was left to cure according to the datasheet, after which the cast was nicked with a surgical blade and opened up by hand. This ensured that the blade never came in contact with the sample. After removing the sample, the casts were split perpendicular to the samples longitudinal axis and scanned at a resolution of 2400 dpi. From these images, showing a cross-sectional imprint of the samples’ midsection, the CSA, thickness, and width of the samples were determined using ImageJ software (U.S. National Institutes of Health, Bethesda, MD, USA) (Scholze et al. 2018). The tensile tests were conducted on a universal testing machine (Z020 equipped with Xforce HP, 500 N Loadcell, both ZwickRoell, Ulm, Germany). Prior to testing, the samples were preconditioned for ten loading–unloading cycles from 0 to 6 N at 0.1 mm/s. The preconditioning load was set at 5% of the F_max_, as determined in preliminary tests. The failure tensile tests were performed at a testing velocity of 0.1 mm/s until a force drop below 95% of maximum force (F_max_) was detected. The force and strain were recorded by testXpert III (ZwickRoell). After testing, the samples were stored at 7 °C and used for CyQuant and DMMB assay. The CSA and the force-strain data were processed in a Matlab routine (R2020a; MathWorks, Natick, MA, USA) to calculate the elastic modulus, the strain at failure, and tensile strength. Due to a section of non-linear behavior before F_max_, the evaluation of stiffness was limited to 0–40% of the maximum force reading. This gave a linear region for each sample to evaluate.

### Statistical analyses

All values were expressed as mean with standard deviation using GraphPad Prism 8 (GraphPad Software Inc., San Diego, CA, USA). Data based on score values (ordinal scaled data, e.g., scoring values) and non-parametric data (e.g., DNA and GAG contents, biomechanical properties) were analyzed using Kruskal–Wallis test followed by Dunn's post hoc test. The Rout test was used to identify outlier. Normality was assessed using a Shapiro–Wilk test. Statistical significance was set at a *p* value of ≤ 0.05.

## Results

### Clinical observations

**Fig. 1:**
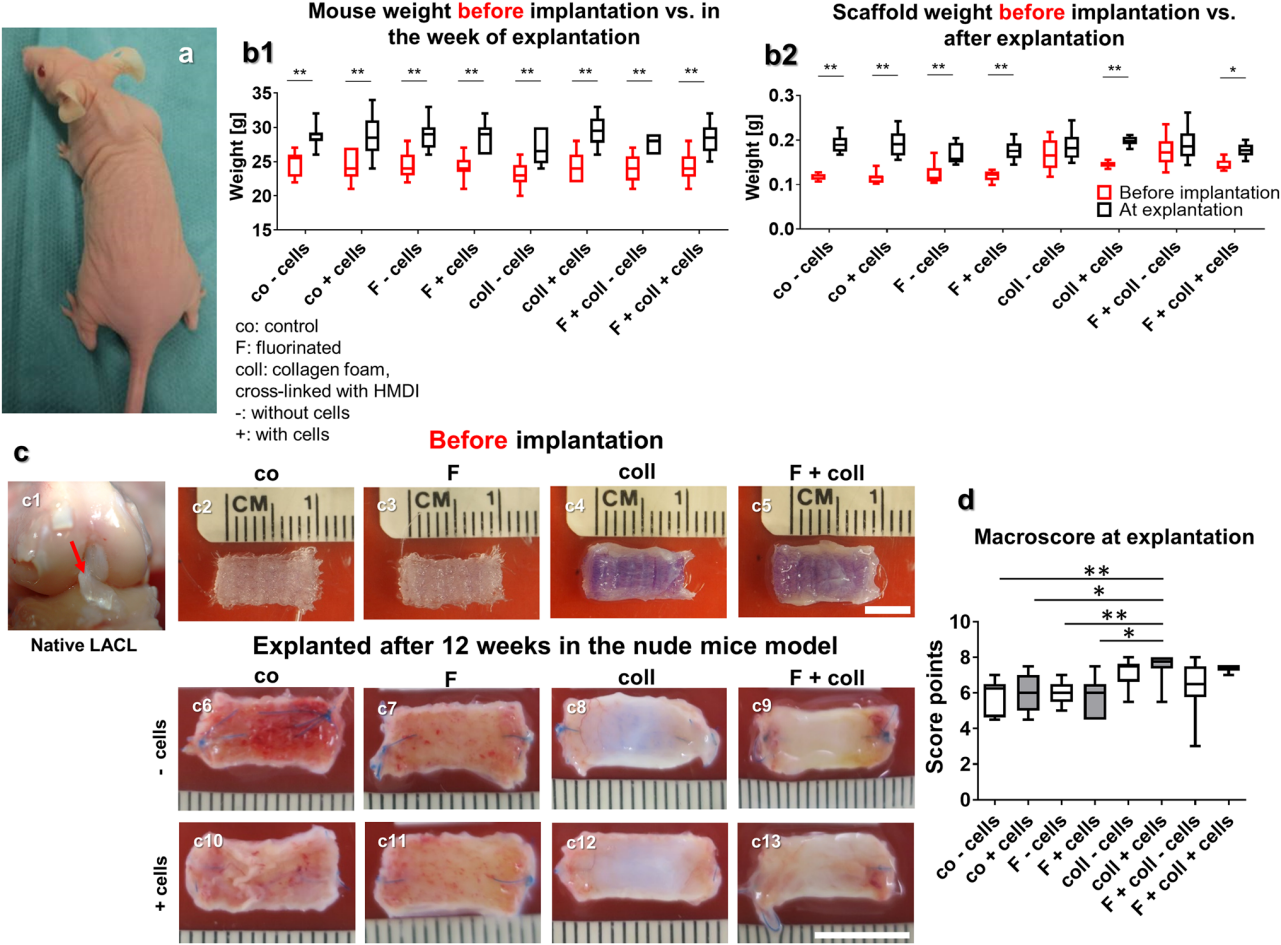
Body weight development of the animals, weight, and macroscopical appearance of the scaffold variants before implantation (after 1 week in vitro) and after explantation after 3 months in vivo. **a1** Mice with a scaffold implanted into the subnuchal region. **b1** Body weight of the mice in the week before scaffold implantation (*red box plots*) and in the week of explantation (*black box plots*). **b2** Scaffold weight (wet weights,* red box plots*) pre-implantation and post explantation (*black box plots*). Macroscopical appearance of the lapine anterior cruciate ligament (LACL) (**c1**,* red arrow*) and the scaffold variants before implantation (**c2–c5**) and after explantation (**c6–c13**). **d** Results of macroscoring of the scaffolds. Co: controls; F: functionalization by gas fluorination; coll: collagen foam cross-linked with HMDI, -: implanted without cells, + : implanted with LACL-derived ligamentocytes, cultured for 1 week on the scaffold before scaffold implantation. *p* values: * < 0.05, ** < 0.01.* Scale bars* 1 cm

The animals showed no change in their behavior following the scaffold implantation, and during the following 12 weeks before scaffold explantation (Fig. 1a). The overall body weight development of the mice during these 12 weeks revealed a continuous increase until the 11th week (supplemental Fig. 1) and did not differ between the groups with different scaffold types implanted (Fig. 1b1) and also not from the expected body weight provided by the supplier (Charles River Laboratories). A significant increase in body weight could be detected in each group compared to that before implantation.

### Scaffold weights, size maintenance, and stability

**Fig. 2:**
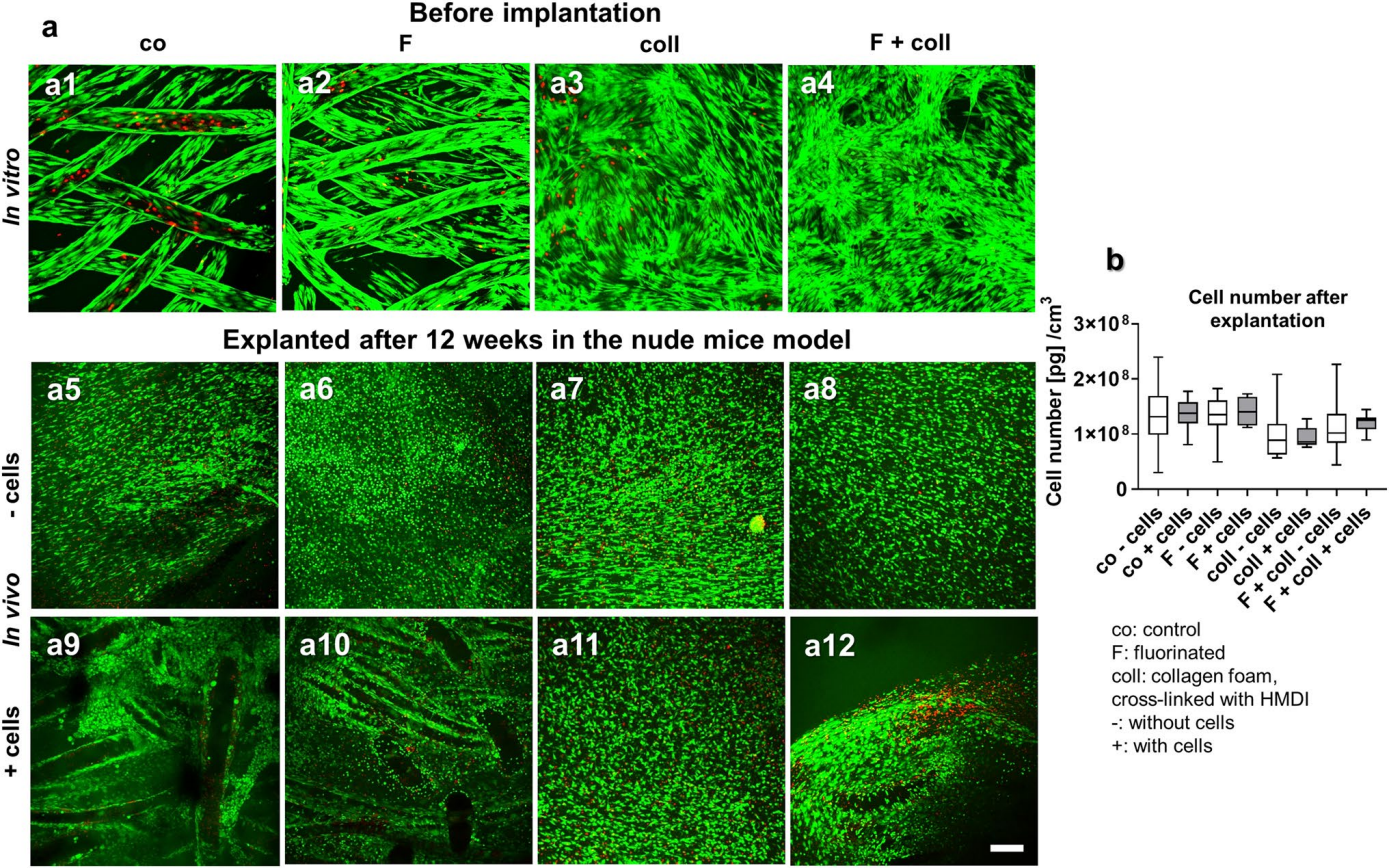
Cell viability and content in the scaffolds before implantation and after explantation after 3 months remaining in vivo. Cell viability in the scaffold variants before implantation after 1 week (**a1–a4**) and after explantation after 3 months in the nude mice model (**a5–a12**), scaffolds implanted without cells (**a5–a8**), and with lapine anterior cruciate ligament (LACL)-derived ligamentocytes, cultured for 1 week on the scaffold before scaffold implantation (**a9–a12**), control (**a1, a5, a9**), F (**a2, a6, a10**), collagen (**a3, a7, a11**) and F + collagen (**a4, a8, a12**). Living cells are stained* green* and dead cells are stained in* red*. **b** Measurement of DNA contents (pg DNA per cm^3^ scaffold) of the scaffold variants after implantation. Co: controls; F: functionalization by gas-phase fluorination; coll: collagen foam cross-linked with hexamethylene diisocyanate (HMDI), -: implanted without cells, + : implanted with LACL-derived ligamentocytes.* Scale bar* 100 µm

The scaffold showed a high porosity visualized by SEM (supplemental Fig. 2). The scaffold weight before implantation ranged between 0.115 ± 0.007 g (control without cells) and 0.16 ± 0.03 g (fluorinated with collagen and cells) and at the time point of explantation between 1.41 ± 0.02 g (fluorinated, pre-seeded LACL ligamentocytes) and 1.91 ± 0.034 g (fluorinated with collagen and preseeded with cells) (Fig. 1b2). Before implantation, scaffolds with collagen foam, irrespective of whether seeded with cells or not, were heavier than controls (not significant) and solely fluorinated scaffolds, but there was no significant difference after implantation (Fig. 1b1, 2). The weights of all scaffold variants were significantly higher following the explantation when compared to their weight before implantation except for the scaffolds supplemented with collagen, irrespectively whether fluorinated and seeded or not. Scaffolds implanted without or with cells, irrespective of whether functionalized or not, generally remained stable and maintained their shape and size (Fig. 2c). However, the pores visible in the scaffolds before implantation were then filled with tissue. A very thin translucent membrane and often some fatty tissue surrounded the implanted scaffolds.

### Macroscopical appearance, size, and shape stability

A macroscopical scoring system (Table 2) was used to detect differences of the macroscopical appearance as well as changes in the size and shape of the explanted scaffolds. As references, the appearance of the native LACL and the scaffolds before implantation were used (Fig. 1c, c1–c5). Collagen supplementation of the scaffolds led generally to a ligament-like appearance resembling the native LACL and accordingly, higher score values. Scaffolds with collagen combined with cells exhibited the most ligamentous impression with significantly higher score values than fluorinated scaffolds without or with LACL ligamentocytes and the control constructs without cells. The gas-phase fluorinated scaffolds with cross-linked collagen foam, but without cells, also had significantly higher score values (Fig. 1c6–c13, d).

### Cell viability of the scaffolds before implantation and after explantation

At the time point of scaffold implantation in the mice subnuchal space, which was respectively 7–8 days after cell-seeding, all scaffolds were covered by mostly viable LACL cells (Fig. 2a1–a4). The scaffold’s functionalization with collagen foam, alone or combined with fluorination, revealed the most densely colonized surface of the scaffold (Fig. 2a3–a4). Immediately after explantation, constructs contained mainly viable cells, with no difference between the different scaffold variants and whether cell seeding was performed before implantation or not (Fig. 2a5–a12).

### DNA content of the scaffold variants

The DNA content was quantified to estimate cell quantities in the scaffold. Despite that, the DNA content did not significantly differ between all investigated scaffold variants implanted without or with ligamentocytes (Fig. 2b). When calling up a previous study where we determined a mean DNA content of 2.1 ± 1.2 µg/cm^3^ in the native LACL (Gögele et al. 2021), the DNA contents in the scaffold variants of this study were substantially higher than that of the native LACL.

### Histology and organ toxicity

**Fig. 3:**
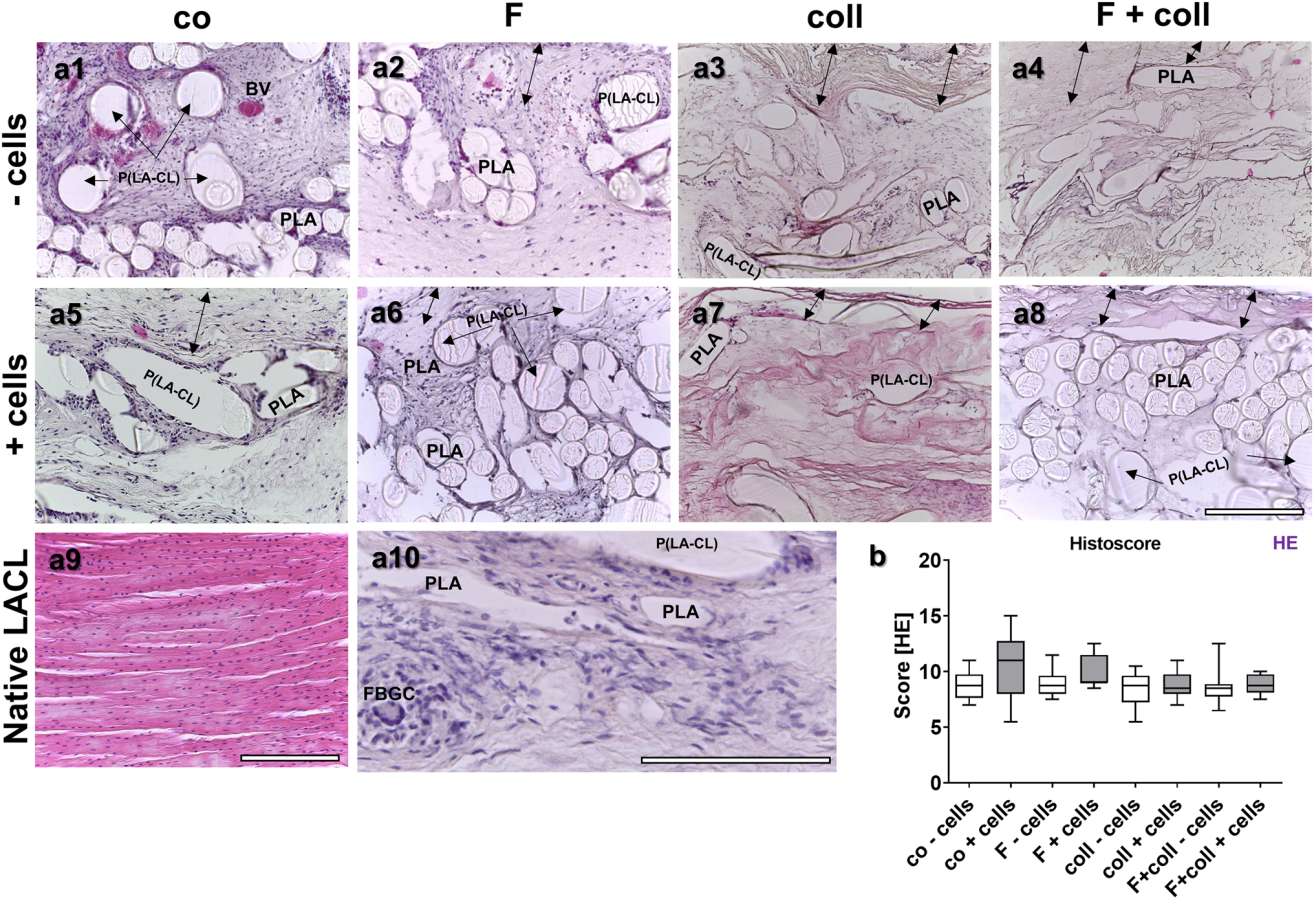
Scaffold histology after explantation after 3 months in vivo depicted by HE staining. Histology of the tissue is shown within the scaffold variants implanted without cells (-cells, **a1–a4**) and with lapine anterior cruciate ligament (LACL)-derived ligamentocytes (+ cells, **a5–a8**). Control (co, **a1, a5**), functionalization by gas-phase fluorination (F, **a2, a6**), collagen foam cross-linked with hexamethylene diisocyanate (HMDI) (coll, **a3, a7**) and F + coll (**a4, a8**). Native LACL (**a9**). Foreign-body giant cell and inflammatory cells visible in a co-cells scaffold variant (**a10,** FBGC). -cells: implanted without cells, + cells: implanted with LACL-derived ligamentocytes.* BV* blood vessel,* PLA* poly lactic acid,* P(LA-CL)* poly(lactic-*co*-ε-caprolactone).* Double-headed black arrows* capsule. In all images, the outer capsule surrounding the scaffold variants can be expected at the upper border.* Scale bar* 200 µm (**a1–a9**), 250 µm (**a10**)

No histopathological changes of the liver, kidney, and spleen were observed, with no presence of monocytes or foreign-body giant cell formation (FBGC) as indicators of inflammation, suggesting no toxic effects induced by the implanted constructs (Supplemental Figs. 3–5). This underlines that all scaffold variants exerted no organ toxicity. Scaffold histology revealed tissue formation within the scaffolds (Fig. 3a1–a9) with no significant differences in the score values between scaffold variants. However, some inflammatory and foreign-body giant cell formation (Fig. 3a10) could be detected in the immediate neighborhood of the fibers, within the scaffolds without collagen foam, whereas those with collagen showed a lower degree of inflammation (Fig. 3). Cells were generally randomly distributed within the constructs. In the collagen foam, there were often only a few cells. However, none of the scaffolds showed cell alignment when compared to the native LACL and also the ECM density was less and not as organized as observed in the native LACL.

### Sulphated glycosaminoglycan content

**Fig. 4:**
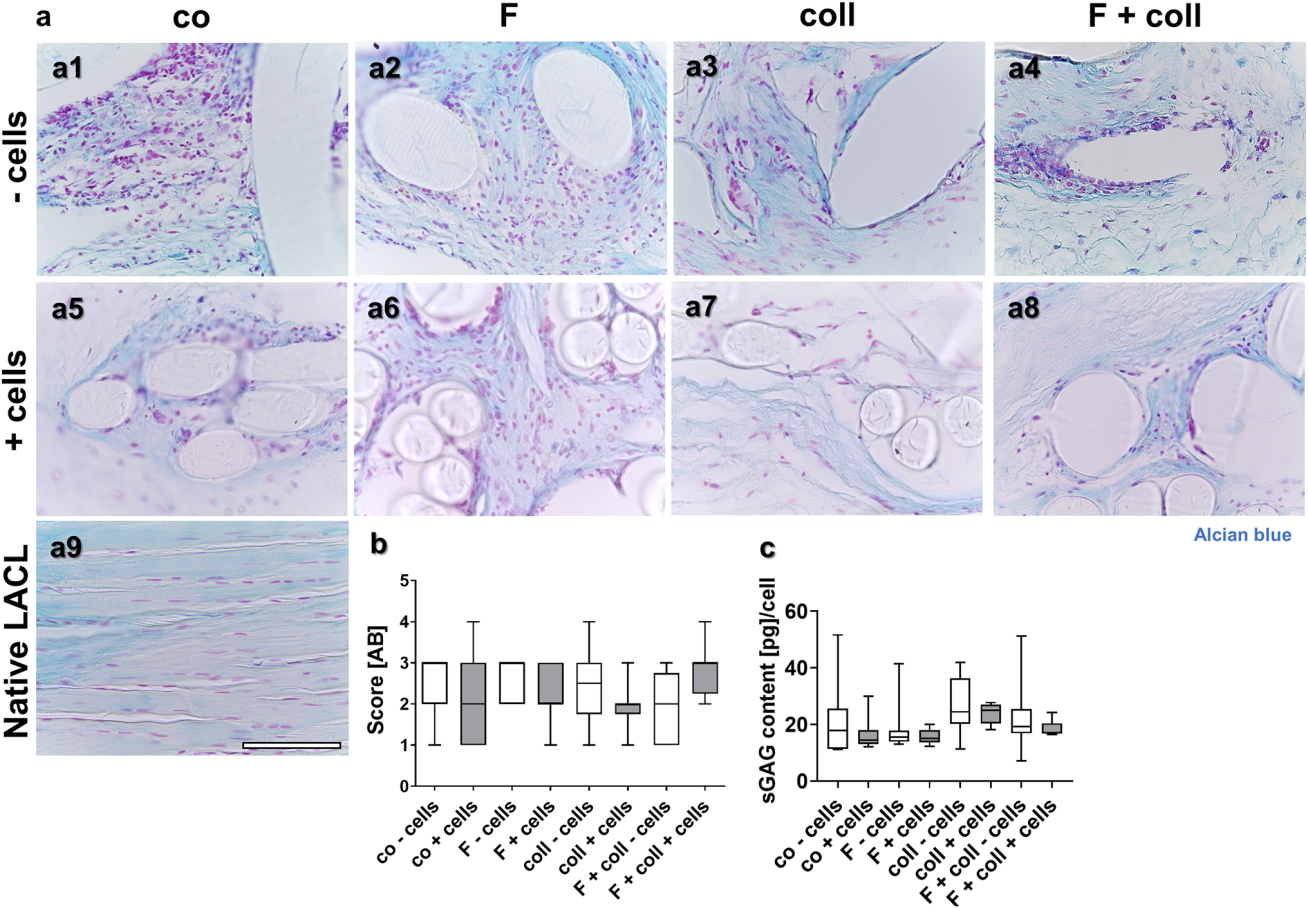
Visualization of sulphated glycosaminoglycans in the different scaffold variants explanted after 3 months in vivo. AB stain to visualize sulphated glycosaminoglycans (sGAGs) of the tissue within the scaffold variants without cells (-cells, **a1–a4**) and with lapine anterior cruciate ligament (LACL)-derived ligamentocytes, cultured for 1 week on the scaffold in vitro before implantation into nude mice for 12 weeks (**a5–a8**), control (co, **a1, a5**), functionalization by gas-phase fluorination (F, **a2, a6**), collagen foam cross-linked with hexamethylene diisocyanate (HMDI) (coll, **a3, a7**) and F + coll (**a4, a8**). AB-stained native LACL (**a9**). **b** Results of AB scoring. **c** sGAG content measured by dimethyl methylene blue (DMMB) assay. -: implanted without cells, + : implanted with LACL-derived ligamentocytes. Kruskal–Wallis test was used for analyses followed by Dunn's post hoc test. *p* values: * < 0.05.* Scale bars* 100 µm (**a1–a9**)

Sulphated glycosaminoglycan (sGAG) deposition was visualized using AB staining (Fig. 4a1–a9) and classified using a scoring system related to the normal staining in the native LACL (Fig. 4a9). The native LACL showed a faint AB staining (Fig. 4a9) and only the interfascicular extracellular matrix (Fig. 4a9) and the enthesis region displayed a focally more intense staining in the fibrocartilage regions (not shown). There were no significant differences between the score values of all scaffold variants investigated (Fig. 4b). In addition, a quantitative assessment of the sGAG content was brought off with the DMMB assay (Fig. 4c). Nevertheless, there was no significant difference in the sGAG content of the different groups, irrespective of whether seeded with cells or not. In comparison to the sGAG content of the native LACL (36.8 ± 12.9 pg/cell) measured in our previous study (Gögele et al. 2021), the sGAG contents of the scaffold variants were generally lower.

### Collagen content and distribution

**Fig. 5:**
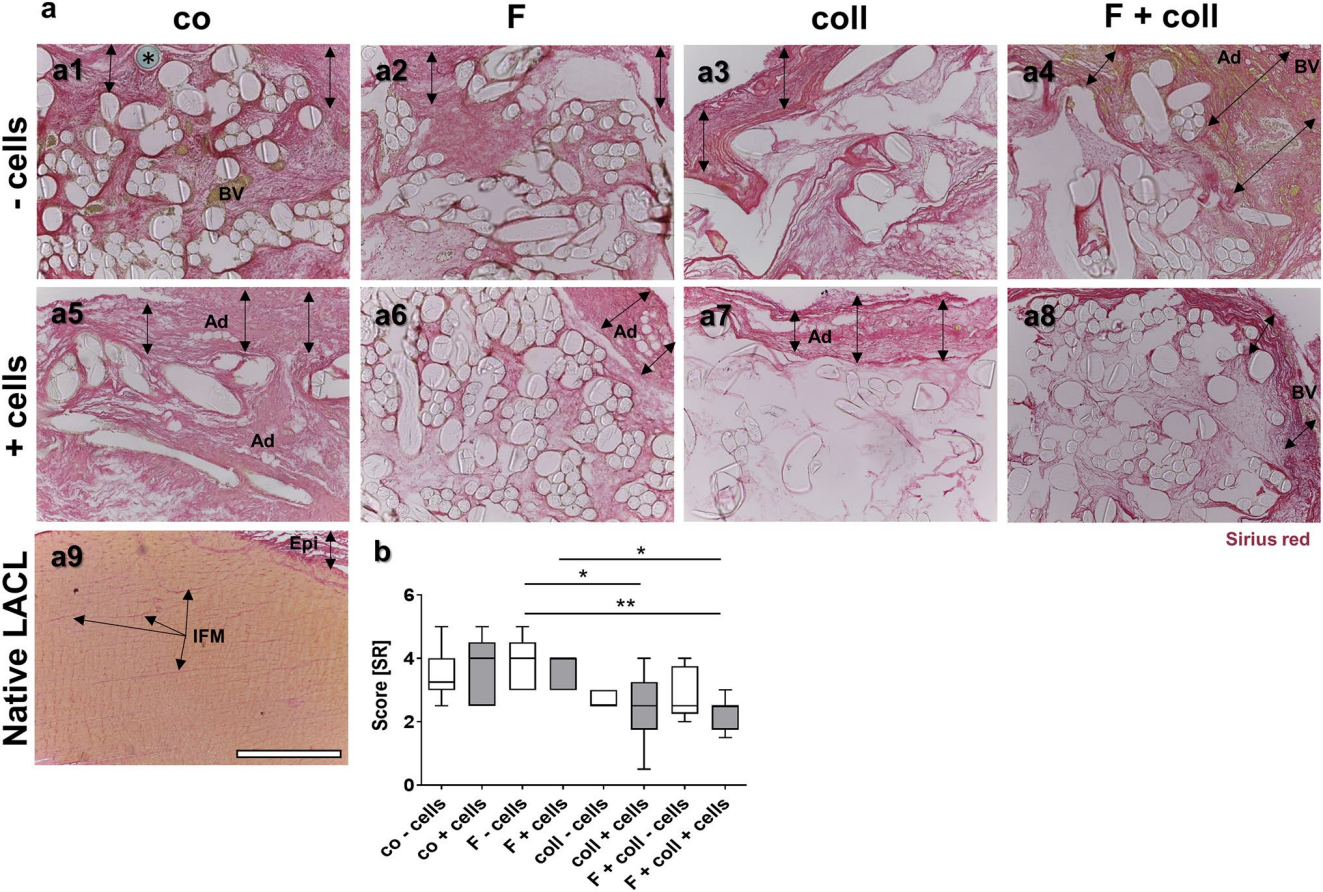
Visualization of collagen in the different scaffold variants explanted after 3 months in vivo. SR stain to visualize collagen organization of the tissue within the scaffold variants without cells (**a1–a4**) and implanted with lapine anterior cruciate ligament (LACL)-derived ligamentocytes, cultured for 1 week on the scaffold in vitro before explanted after 12 weeks in vivo (**a5–a8**), co (**a1, a5**), F (**a2, a6**), coll (**a3, a7**) and F + coll (**a4, a8**). Native LACL (**a9**).* Double-headed black arrows* indicate an envelope rich in collagen surrounding the scaffold. **b** Results of Sirius red scoring. -: implanted without cells, + : implanted with LACL-derived ligamentocytes.* Ad* adipocytes,* BV* blood vessels,* Epi* epiligament,* IFM* interfascicular matrix. *prolene thread used for scaffold fixation. Kruskal–Wallis test was used for analyses followed by Dunn's post hoc test. *p* values: * < 0.05.* Scale bars* 400 µm (**a1–a9**)

Collagen has been visualized in all scaffold variants using SR staining (Fig. 5a1–a9). In most samples, there was a more intensely stained envelope around the construct (double headed arrows) containing a more faintly stained tissue within the fiber network. The scaffolds functionalized with collagen either combined with or without fluorination revealed lesser collagen stain inside. The native LACL showed a homogenous more orange SR stain and densely packaged fiber bundles. The difference in staining was clearly detectable by polarization microscopy where the native LACL was much more yellow, indicating organized type I collagen than the tissue in the scaffold variants (supplemental Fig. 6). The latter show only focal yellow areas. Only the epiligament and interfascicular ECM was stained in red (Fig. 5a9, Epi, IFM). SR scoring (Fig. 5b) revealed significantly higher score values for fluorinated scaffolds without cells compared to those with collagen foam pre-seeded with cells irrespective of fluorination. In addition, the scaffolds with cells and fluorination had significantly higher score values than those with collagen foam and fluorination. In the envelope of several samples, emigrated adipocytes could be visualized (Fig. 5a4–a7).

**Fig. 6:**
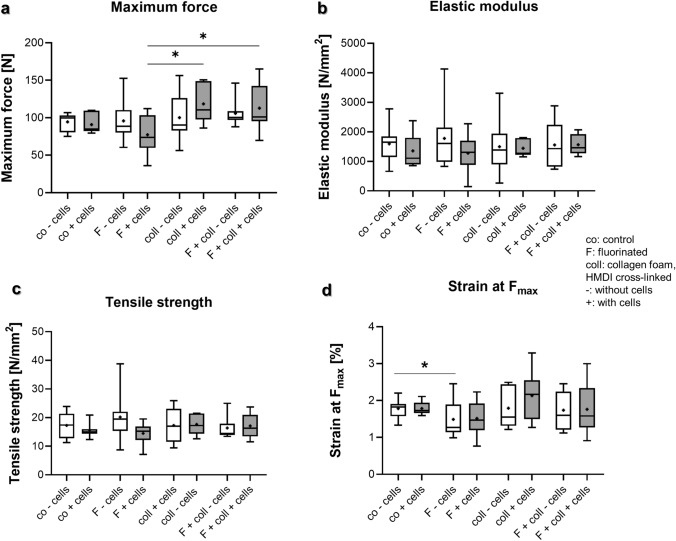
Biomechanical analyses of scaffold variants explanted after 3 months in vivo. **a** Maximum force, **b** elastic modulus, **c** tensile strength, **d** strain at maximum force. *Co* controls, *F* functionalization by gas fluorination, *coll* collagen foam cross-linked with hexamethylene diisocyanate (HMDI), -: implanted without cells, + : implanted with lapine anterior cruciate ligament (LACL)-derived ligamentocytes, cultured for 1 week on the scaffold before scaffold implantation. Normality was assessed using a Shapiro–Wilk test and groups compared using Kruskal–Wallis test. *p* values: * < 0.05

### Biomechanical analyses

The biomechanical parameters of the scaffold variants explanted after 3 months either cell-free or seeded with cells did not significantly differ. Hence, the scaffolds without and with cells, were pooled for the comparison of scaffolds before implantation into the nude mice and 3 months later, after explantation.

### Comparison of biomechanical parameters after 12 weeks in vivo

Statistical comparison of maximum force yielded significantly lower values for fluorinated scaffolds (77.2 ± 27.0 N) when compared to scaffolds with collagen foam (118.4 ± 25.8 N; *p* = 0.02) and those fluorinated and supplemented with collagen foam (112.8 N; *p* = 0.04), respectively (Fig. 6a). Elastic modulus and tensile strength did not differ between the explanted samples (Fig. 6b, c). However, the elastic modulus gave for F ± cells, coll-cells and F + coll-cells much broader variations when compared to the other groups. Similarly, tensile strength and strain at F_max_ yielded similar and statistically non-different results to the mechanical values (Fig. 6c, d).

### Comparison of biomechanical parameters of scaffolds before implantation and after explantation

**Fig. 7:**
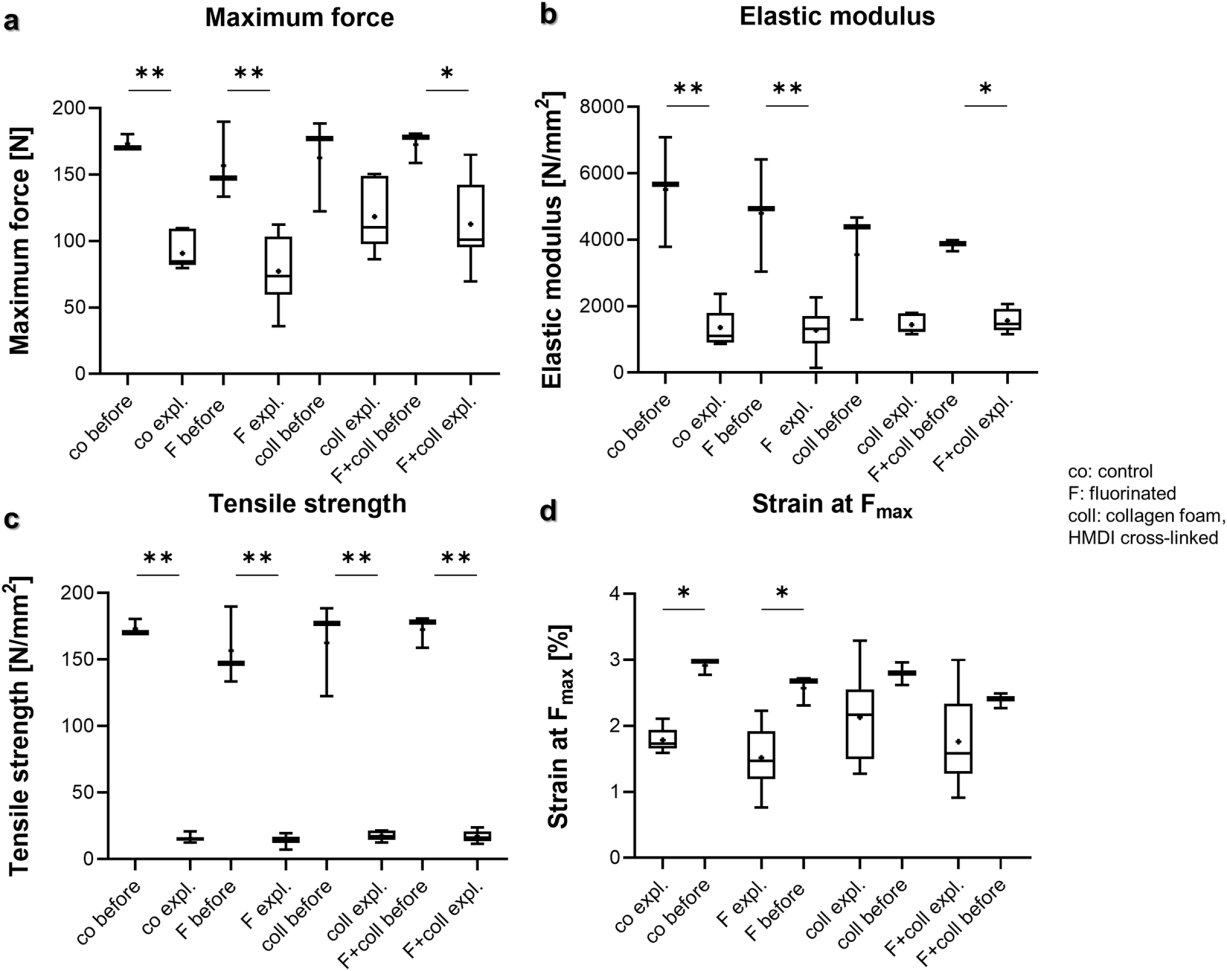
Biomechanical analyses of scaffold variants explanted after 3 months in vivo compared to those before implantation (cultured 1 week in vitro).** a** Maximum force, **b** elastic modulus, **c** tensile strength, **d** strain at maximum force. Unseeded scaffolds and those seeded with cells were pooled. *Co* controls, *F* functionalization by gas-phase fluorination; *coll* collagen foam cross-linked with hexamethylene diisocyanate (HMDI). Normality was assessed using a Shapiro–Wilk test and groups compared using Kruskal–Wallis test. *p* values: * < 0.05, ** < 0.01, *** < 0.005

In most cases, maximum force, elastic modulus, tensile strength, strain at F_max_ differed significantly between the scaffolds before implantation and the samples explanted after 12 weeks (in vivo) (Fig. 7). There was a general trend of the data before implantation to give higher values than the in vivo data 12 weeks later. For maximum force, the values for control scaffolds before implantation were significantly higher than those for the same scaffolds after additional 12 weeks in vivo (173.2 ± 6.3 vs. 90.8 ± 13.0 N; *p* = 0.003), the fluorinated scaffolds cultured only 1 week in vitro before implantation had higher values than those implanted for additional 12 weeks in vivo (156.8 ± 29.4 vs. 77.2 ± 27.0 N; *p* = 0.003). In a similar manner, fluorinated scaffolds supplemented with collagen had higher maximum forces after 1 week in vitro before implantation than those explanted after 12 weeks in vivo (172.6 ± 12.0 vs. 112.8 ± 30.3 N; *p* = 0.05) (Fig. 7a).

Elastic modulus was similarly higher in the controls cultured only in vitro before implanted into nude mice when compared to the same samples explanted after 3 months in vivo (5511.9 ± 1653.8 vs. 1354.5 ± 556.0 N/mm^2^; *p* = 0.003) and also higher in the fluorinated samples before being implanted than in the same samples implanted for 3 months into nude mice (1277.3 vs. 696.8 N/mm^2^; *p* = 0.005). There was also a significant difference between the values for scaffolds fluorinated and supplemented with collagen foam (3840.9 ± 169.9 vs. 1565.8 ± 351.1 N/mm^2^; *p* = 0.04) before implantation and after explantation (Fig. 7b). Tensile strength was higher in the controls before being implanted vs. those after 3 months in vivo (173.2 ± 6.3 vs. 15.6 ± 2.6 N/mm^2^; *p* = 0.003) and also the fluorinated samples differed significantly before implantation vs. after explantation after 3 months in vivo (156.8 ± 6.3 vs. 14.5 ± 4.1 N/mm^2^; *p* = 0.003). There was also a significant difference between the samples supplemented with collagen before implantation vs. those explanted from nude mice (162.6 ± 35.4 vs. 17.6 ± 3.4 N/mm^2^; *p* = 0.03) and between the fluorinated samples supplemented with collagen before implantation vs. those explanted from nude mice (172.6 ± 12.0 vs. 17.1 ± 4.2 N/mm^2^; *p* = 0.008), respectively (Fig. 7c). Of note, the values achieved for both elastic modulus and tensile strength before implantation averaged a multiple of the values observed in the scaffolds explanted from mice. Similarly, strain at F_max_ was higher for the non-functionalized scaffolds before implantation vs. the same variant after explantation (2.9 ± 0.1 vs. 1.8 ± 0.2%; *p* = 0.02) and fluorinated scaffolds before implantation vs. those cultured for 3 months (2.6 ± 0.2 vs. 1.5 ± 0.5%; *p* = 0.02) (Fig. 7d).

## Discussion

This study provided biomechanical characteristics and proved for the first time the in vivo formation of an ECM-rich connective tissue within the functionalized embroidered scaffold variants with a high collagen and a low sGAG content as expected in ligaments. The scaffolds with collagen supplementation had a macroscopical ligament-like appearance. The scaffold variants remained stable in size and shape over the 3-month implantation time and showed high cell viability. The scaffold variants used in the present in vivo study have been previously tested in vitro, showing their high cytocompatibility (Gögele et al. 2020a). In the present in vivo study, no organ toxicity of the embroidered and functionalized LACL scaffolds could be demonstrated. All tested scaffold variants indicated a high cell viability on the scaffolds after explantation and no detectable systemic effects on the animals, particularly, no toxic effects on metabolization, immune and excretion organs including the liver, spleen, and kidneys. Supporting these findings, animal weight development and behavior remained unaffected throughout the entire study. The previous in vitro studies indicated that the functionalization with gas-phase fluorination and a cross-linked collagen foam supported cell spreading and adherence on the scaffold (Gögele et al. 2021, 2020b). This preceding observation could be substantiated and expanded in the here given experiments. It was documented by a higher confluence of vital LACL ligamentocytes on functionalized scaffolds after 7 days culturing in vitro before implantation into nude mice. The weight of most scaffold variants increased significantly after the in vivo incubation suggesting tissue formation and ingrowth. It was not surprising that before implantation the scaffolds supplemented with collagen foam showed higher weights probably due to the higher water binding capacity. However, this difference was diminished following implantation. There was also no clear scaffold encapsulation seen, which is very often observed but unwanted when using biomaterials (Sivam et al. 2022). There was only a thin, translucent, shiny, and glossy membrane that covered the explants. Histologically, a collagen-rich envelope was detected, particularly in the scaffolds with collagen, which most probably represented the collagen foam covering the scaffold surface. Macroscopically, a ligament-like tissue formation was observed, particularly in the scaffolds functionalized with collagen and seeded with cells.

Collagen is the predominant formed ECM component in ligament tissues. An intense collagen staining could be detected in all scaffold variants underlining their content of collagen. However, there was less collagen detectable in the inner parts of constructs with collagen functionalization when compared to the scaffolds without collagen foam, as indicated by the SR staining. This may have been influenced by the lower cell content producing novel collagen. Nevertheless, the cell number was higher than that reported in a preceding study for the LACL (Gögele et al. 2021). This might indicate that the maturation of the tissue in the scaffold variants after 12 weeks in vivo is not as advanced as to be expected in the native ligament. The scaffold variants, not only those supplemented with collagen foam, had a collagenous envelope stained more intensively red. This structure resembled somehow the epiligament of the native LACL. The native LACL stained for comparison had a less intense red color but showed a highly saturated orange staining, reflecting the densely packed type I collagen fibrils. Only the LACL epiligament and the interfascicular ECM showed a red color comparable to that in the scaffolds, particular their envelopes. Hence, one has to conclude that the collagen organization in the scaffolds is not as mature as in the LACL.

On the contrary, inflammation was more obvious in constructs without collagen functionalization, probably due to the direct contact between cells and scaffold fibers or the compensation of acidic by-products of degrading PLA in these non-functionalized scaffolds and the better access of the immune cells since the collagen foam formed a barrier for them. Inflammation was mostly fiber-associated with FBGC. Macrophage-mediated FBGC reaction against PLA in healing tendons has been reported previously (Liu et al. 2017). Likewise, polycaprolactone (PCL) was associated with inflammation in a rat model of ACL substitution (Kawakami et al. 2021), and with few FBGC (Rashid et al. 2020; Reifenrath et al. 2020). Fiber alignment influenced this inflammatory response of tendon fibroblasts to PCL (Schoenenberger et al. 2018). The study given here used a combination of P(LA-CL) with PLA. The P(LA-CL) monofilament is a copolymer of PLA and PCL with a ratio of 75:25 (Tomihata et al. 1998), suggesting that the effect of PLA most probably dominates. This combination showed favorable biomechanical properties (Hahn et al. 2019). Hence, it is not surprising that there was no major difference in the degree of macrophage and FBGC accumulation at either the P(LA-CL) fibers (larger diameter fibers) and PLA with smaller fiber diameters. Immune cells, particularly macrophages, are well known to contribute to tendon healing (Schulze-Tanzil et al. 2022) and hence, macrophage polarization has to be considered. ECM components such as collagen (Kajahn et al. 2012), but also scaffold material, might influence macrophage polarization from pro-inflammatory M1 to anti-inflammatory M2 type, which is more favorable for healing processes in tendons (Schulze-Tanzil et al. 2022). Due to this observation, a cell-mediated fiber degradation should be considered. Nevertheless, the increase in DNA as an indicator for higher cell contents did not reach a level of significance. In agreement with the hypothesis of the onset of cell-mediated scaffold degradation, the biomechanical analyses indicated that the scaffolds seeded for only 1 week in vitro had significantly higher biomechanical stability than scaffolds precultured for a similar time in vitro and then explanted after 12 weeks in vivo. In this regard, one has to consider that the scaffolds explanted from the mice are not only exposed for a one-fold longer time to cells but also to biomechanical and diverse other systemic stimuli. This process might be weaker in the samples with collagen foam since in contrast to the other variants, there was no significant difference between the samples with collagen foam before implantation and those explanted from nude mice in regard to biomechanical properties including the maximum force, elastic modulus, and strain at F_max_. In scaffold samples with collagen, lesser collagen deposition was observed by Sirius red stain, but significantly greater mechanical resistance. It seems as if the presence of collagen might inhibit further collagen synthesis but possibly also the release of degradative enzymes such as matrix metalloproteinases. Nevertheless, we think that the presence of inflammatory cells and FBGCs contributes substantially to biomechanical weakening of the fibers.

However, the fibers of all scaffold variants showed histologically no signs of damage and had still a smooth fiber surface. Hahn et al. (2019) demonstrated that the same scaffolds, both seeded with ligamentocytes and unseeded, did not show major degradation after 14 and 28 days of in vitro culture. We do not know whether the lapine cells seeded on the scaffolds indeed survive and we could not test it in this study. In this previous study, the biomechanical behavior of degrading matrices with and without cells showed no significant differences (Hahn et al. 2019) and was within the range of the native LACL as determined by Panjabi et al. (2001). Nevertheless, the testing procedure applied in this former study (Hahn et al. 2019) did not include a processing step into a dog-bone shaped structure before measurement, and hence, probably led to higher biomechanical values. The mentioned processing was performed to allow a scaffold failure at a defined and comparable point of the scaffold. The calculated parameters except for maximum force were related to the cross-sectional area, but nevertheless, tailoring might have weakened the embroidered scaffold structure since some loops of the embroidery are indeed destroyed. The same observation that cell seeding before implantation did not increase scaffold degradation could be confirmed by the fact that in the present study, no significant biomechanical differences could be detected between the same scaffolds either implanted with or without cells. This suggests that the collagen foam and ECM components somehow cover potential immunogenic epitopes on the fibers or neutralize acidic degradation products of them. However, the thread materials used for the embroidery served already since years as medical devices. Important ECM components in connective tissues are sGAGs, which are physiologically only weakly expressed in ligaments except for the enthesis part. The score values of AB staining showed no differences on a significant level. In agreement with this staining, the sGAG content measured by the DMMB assay was low in the explanted scaffolds with no significant difference detected between the different scaffold variants and we guess that longer implantation time is needed. Nevertheless, the sGAG content in the scaffolds remained as visualized by the staining and the assay under the level to be expected in the native LACL (Gögele et al. 2021). However, the tissue density within the scaffold variants did not achieve the degree observed in the native LACL and the embedded cells were not aligned in the typical parallel order known for the LACL and demonstrated in histological staining of the LACLs shown in this study. Due to the zig-zag embroidery pattern of the scaffolds, the fibers had only a limited uniaxial alignment. To promote this, probably more time, an enhanced mechanical provocation, or a substructure in micro- or nanoscale on the fibers would be necessary. Despite that the scaffold variants were fixed to the autochthonous muscle of the mice to create a dynamic model, the forces applied in this position might not be high enough for tissue training. Hence, using a heterotopic implantation model here in contrast to applying an orthotopic approach in the knee joint remains a limitation of the present study. Nevertheless, this model allows a broader screening than orthotopic models. The subpopulation of immune cells (such as M1 and M2 polarization of macrophages) was not further investigated here since the focus of this study was on tissue formation and biomechanical properties.

## Conclusions

For all P(LA-CL)/PLA scaffold variants tested in this study in vivo, no organ toxicity, size and shape stability, as well as a content of viable and ECM producing fibroblasts have been confirmed*.* The results, especially for the gas-phase fluorination as a completely novel functionalization strategy, present a new approach for ACL reconstruction scaffolds. None of the functionalization strategies impaired biomechanical stability after 3-month in vivo following the applied biomechanical testing protocol in a dog-bone shape. The slight inflammatory cell response to the scaffold fibers might be modulated by collagen supplementation. Collagen supplementation led to constructs that had macroscopically a typical white and glossy ligament-like appearance significantly superior to variants without collagen. However, histology indicated that the tissue formed during the observation time is still not mature. This suggests that longer in vivo tests and probably an ACL reconstruction model should follow to explain at which time the neo-tissue formed will be mature.

## Supplementary Information

Below is the link to the electronic supplementary material.

Supplementary file1 Supplementary Figure 1: Body weight development of mice after implantation of the different scaffold variants until explantation. co, controls (black: without (-) cells, blue: with (+) cells); F, functionalization by gas-phase fluorination (green); coll, collagen foam cross-linked with HMDI (red); collagen foam combined with gas-phase fluorination (yellow) (TIF 669 KB)

Supplementary file2 Supplemental figure 2: Visualization of the embroidered (P(LA-CL))/PLA scaffold structure at different magnifications by SEM. b: White double-headed arrow: loading direction for the biomechanical experiments. Scale bars 200 µm (a,b), 100 µm (c) (TIF 4080 KB)

Supplementary file3 Supplemental figure 3: Histopathology of the liver of mice 3 months after implantation of the different scaffold variants, depicted by HE staining. Scaffolds without cells (-cells, a1–d2) and implanted with LACL-derived ligamentocytes, cultured for one week on the scaffold before scaffold implantation (+cells, a3–d4). a: co, controls; b: F, functionalization by gas-phase fluorination; c: coll, collagen foam cross-linked with HMDI; d: collagen foam combined with gas-phase fluorination. Scale bars 100 µm (a1–d1, a3–d3), 50 µm (a2–d2, a4–d4). (TIF 11679 KB)

Supplementary file4 Supplemental figure 4: Histopathology of the spleen of mice 3 months after implantation of the different scaffold variants, depicted by HE staining. Scaffolds without cells (-cells, a1–d2) and seeded with LACL-derived ligamentocytes, cultured for one week on the scaffold before scaffold implantation (+cells, a3–d4). a: co, controls; b: F, functionalization by gas-phase fluorination; c: coll, collagen foam cross-linked with HMDI; d: collagen foam combined with gas-phase fluorination. Scale bars 100 µm (a1–d1, a3–d3), 50 µm (a2–d2, a4–d4). (TIF 9844 KB)

Supplementary file5 Supplemental figure 5: Histopathology of the kidney of mice 3 month after implantation of the different scaffold variants, depicted by HE staining. Scaffolds without cells (-cells, a1–d2) and seeded with LACL-derived ligamentocytes, cultured for one week on the scaffold before scaffold implantation (+cells, a3–d4). a: co, controls; b: F, functionalization by gas-phase fluorination; c: coll, collagen foam cross-linked with HMDI; d: collagen foam combined with gas-phase fluorination. Scale bars: 100 µm (a1–d1, a3–d3), 50 µm (a2–d2, a4–d4: the renal cortex is shown at larger magnification). (TIF 11542 KB)

Supplementary file6 Supplemental Figure 6: Visualization of collagen by polarization microscopy in the different scaffold variants explanted after 3 months in vivo and stained with Sirius red (SR). SR stain to visualize collagen organization of the tissue within the scaffold variants without cells (a1–a4) and implanted with lapine anterior cruciate ligament (LACL)-derived ligamentocytes, cultured for one week on the scaffold in vitro before explanted after 12 weeks in vivo (a5–a8), co (a1, a5), F (a2, a6), coll (a3, a7) and F + coll (a4, a8). Native LACL (a9). Scale bars 100 µm (a1–a9). (TIF 11087 KB)

## Abbreviations

AB: Alcian blue
ACL: Anterior cruciate ligament
CLSM: Confocal laser scanning microscopy
co: Unfunctionalized control constructs
coll: Collagen foam cross-linked with HMDI functionalization of the constructs
CSA: Cross-sectional area
DAPI: 4′,6′-Diamidino-2-phenylindol
dist: Distilled
DMMB: Dimethylmethylene blue
dpi: Dots per inch
ECM: Extracellular matrix
EDTA: Ethylenediaminetetraacetic acid
ETOH: Ethanol
F: Gas-phase fluorination of the constructs
FBGC: Foreign-body giant cells
FBS: Fetal bovine serum
F + coll: Gas fluorination combined with collagen foam cross-linked with HMDI of the constructs
FDA: Fluorescein diacetate
F_max_: Maximum force
HBSS: Hank’s balanced salt solution
HE: Hematoxylin/eosin
HMDI: Hexamethylene diisocyanate
L: Lapine
LACL: Lapine anterior cruciate ligament
nondist: Nondistilled
PBE: Phosphate-buffered EDTA
PBS: Phosphate-buffered saline
PCL: Polycaprolactone
P(LA-CL): Poly(L-lactide-co-ε-caprolactone)
PFA: Paraformaldehyde solution in PBS
PI: Propidium iodide
PLA: Polylactic acid
RT: Room temperature
s.c.: Subcutaneous(ly)
SD: Standard deviation
sGAG: Sulphated glycosaminoglycan
SR: Sirius red
TBS: TRIS buffered saline
