# Supplementary figures and images for: In vivo ligamentogenesis in embroidered poly(lactic-co-ε-caprolactone) / polylactic acid scaffolds functionalized by fluorination and hexamethylene diisocyanate cross-linked collagen foams
Source: Histochem Cell Biol. 2022 Oct 29;159(3):275–92. doi: 10.1007/s00418-022-02156-3 (PMC10006054; doi:10.1007/s00418-022-02156-3)

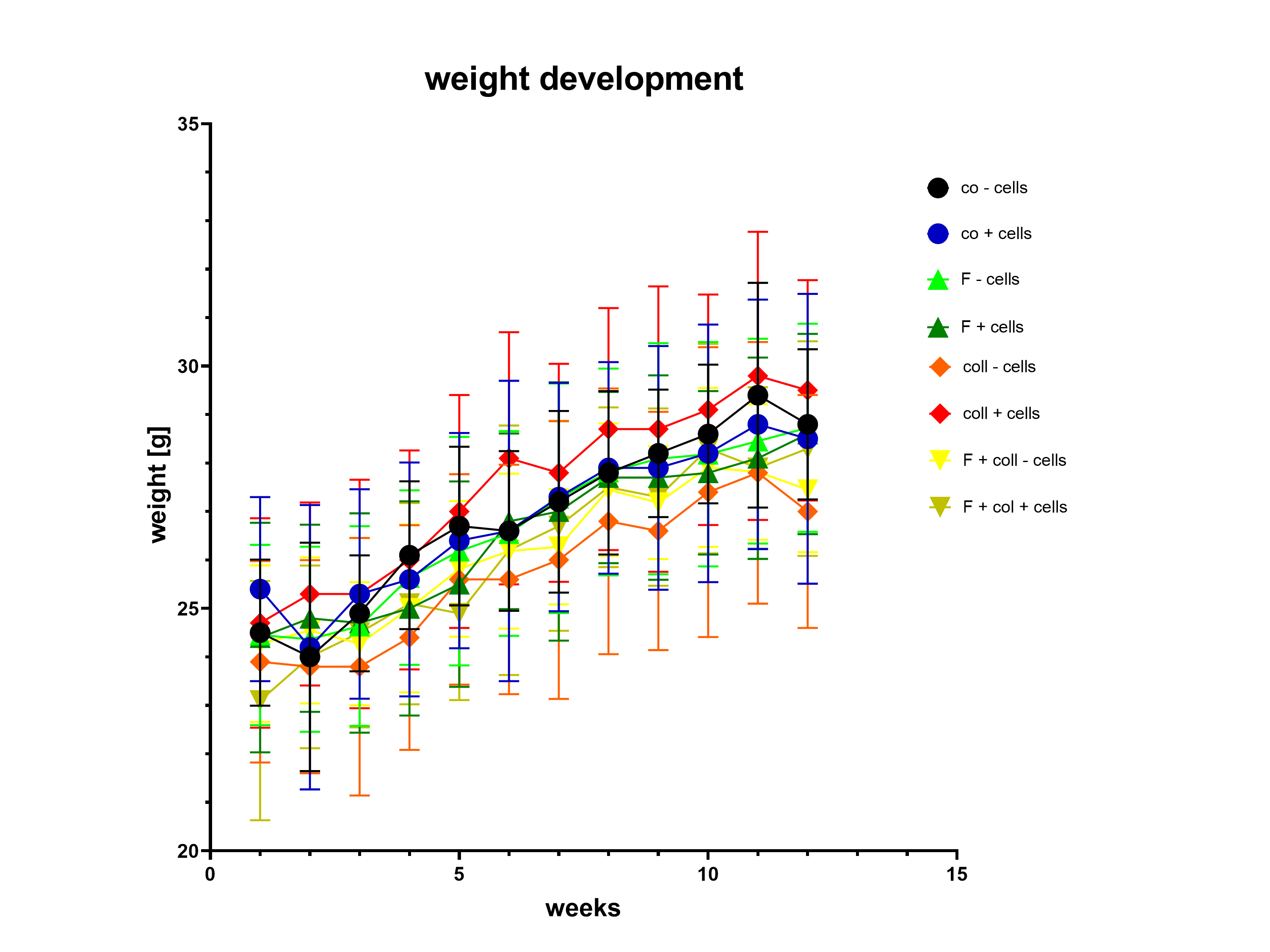

Supplement: Supplementary file 1 — Supplementary file1 Supplementary Figure 1: Body weight development of mice after implantation of the different scaffold variants until explantation. co, controls (black: without (-) cells, blue: with (+) cells); F, functionalization by gas-phase fluorination (green); coll, collagen foam cross-linked with HMDI (red); collagen foam combined with gas-phase fluorination (yellow) (TIF 669 KB) [file 418_2022_2156_MOESM1_ESM.tif]

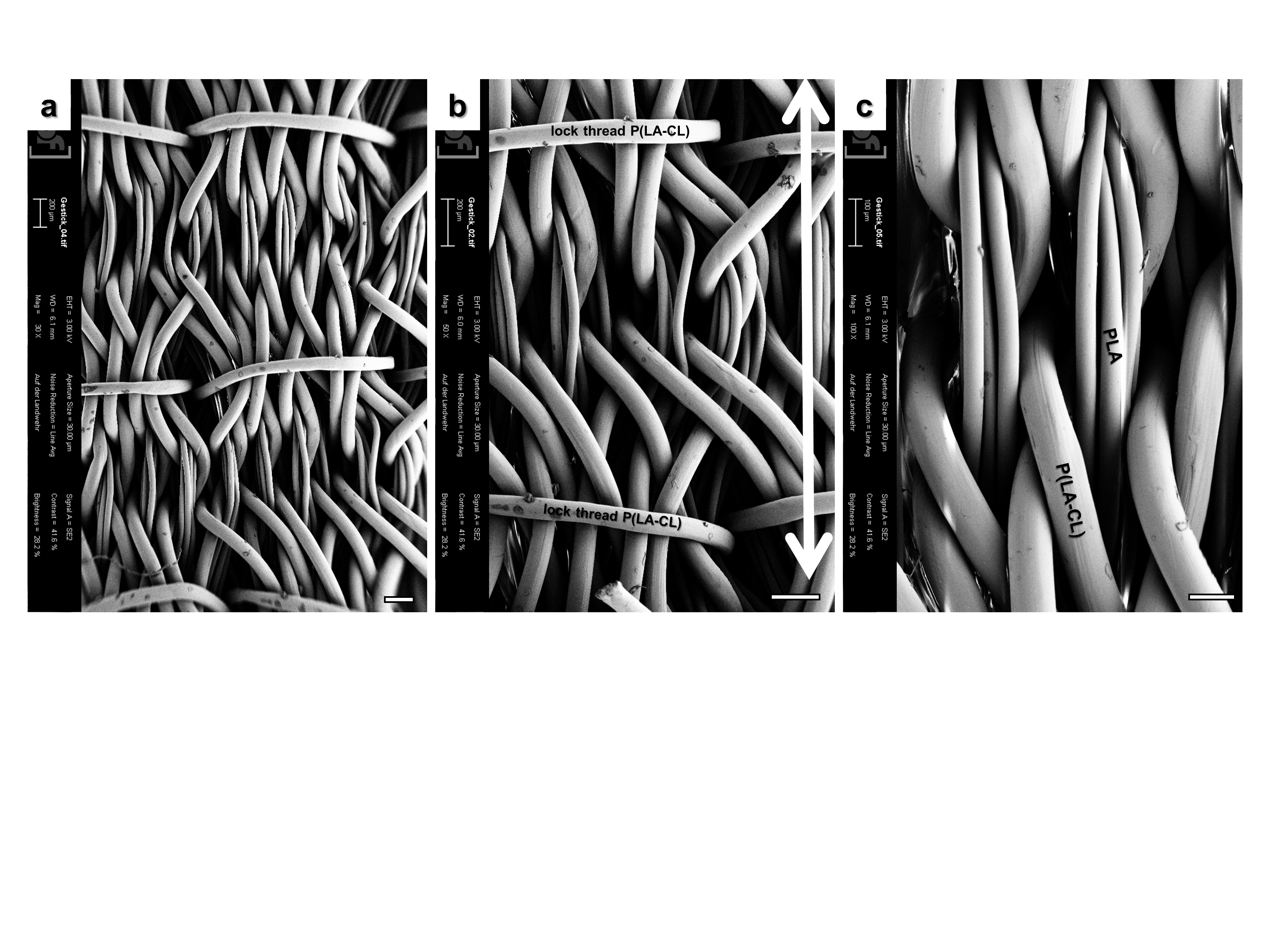

Supplement: Supplementary file 2 — Supplementary file2 Supplemental figure 2: Visualization of the embroidered (P(LA-CL))/PLA scaffold structure at different magnifications by SEM. b: White double-headed arrow: loading direction for the biomechanical experiments. Scale bars 200 µm (a,b), 100 µm (c) (TIF 4080 KB) [file 418_2022_2156_MOESM2_ESM.tif]

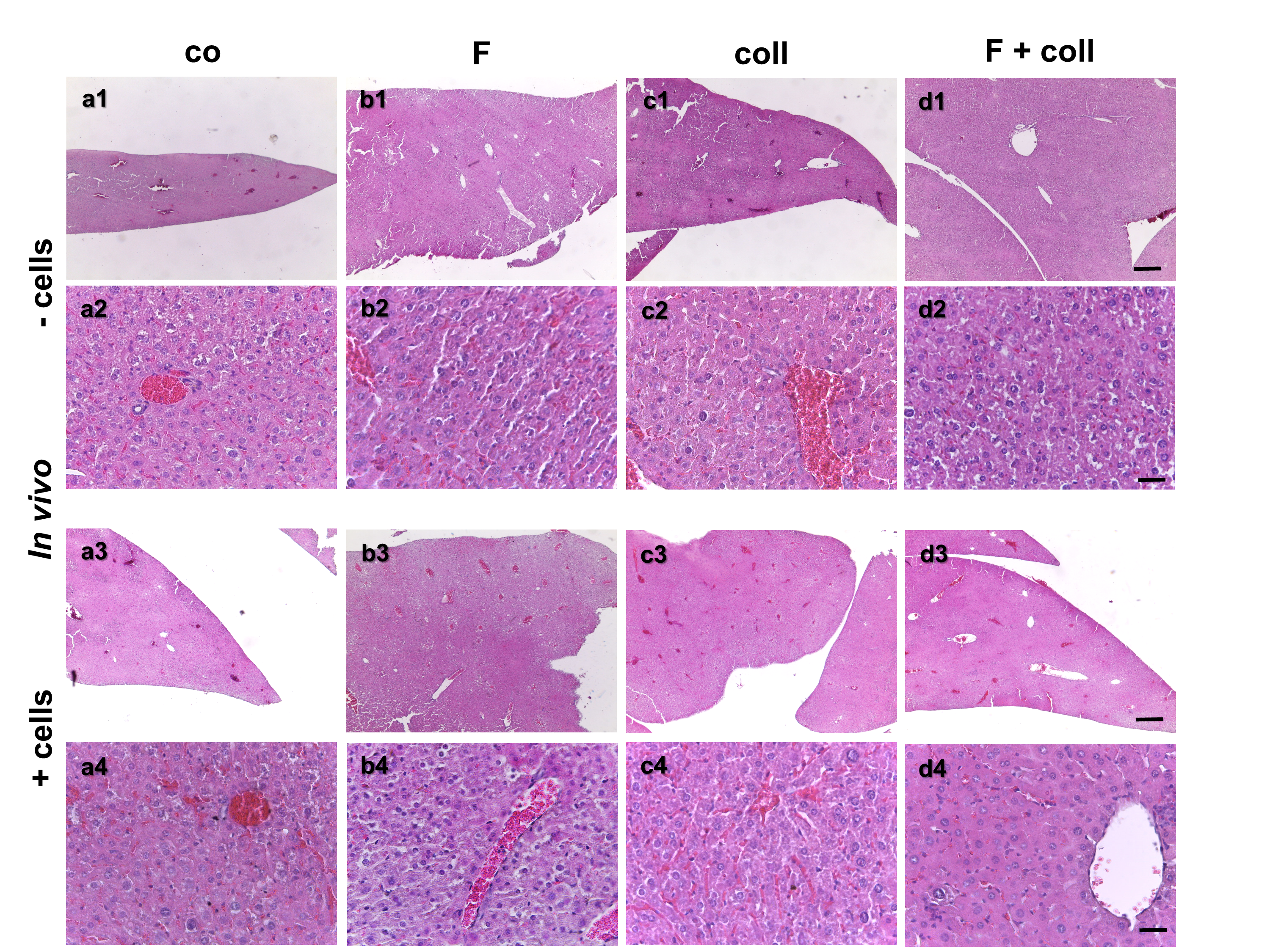

Supplement: Supplementary file 3 — Supplementary file3 Supplemental figure 3: Histopathology of the liver of mice 3 months after implantation of the different scaffold variants, depicted by HE staining. Scaffolds without cells (-cells, a1–d2) and implanted with LACL-derived ligamentocytes, cultured for one week on the scaffold before scaffold implantation (+cells, a3–d4). a: co, controls; b: F, functionalization by gas-phase fluorination; c: coll, collagen foam cross-linked with HMDI; d: collagen foam combined with gas-phase fluorination. Scale bars 100 µm (a1–d1, a3–d3), 50 µm (a2–d2, a4–d4). (TIF 11679 KB) [file 418_2022_2156_MOESM3_ESM.tif]

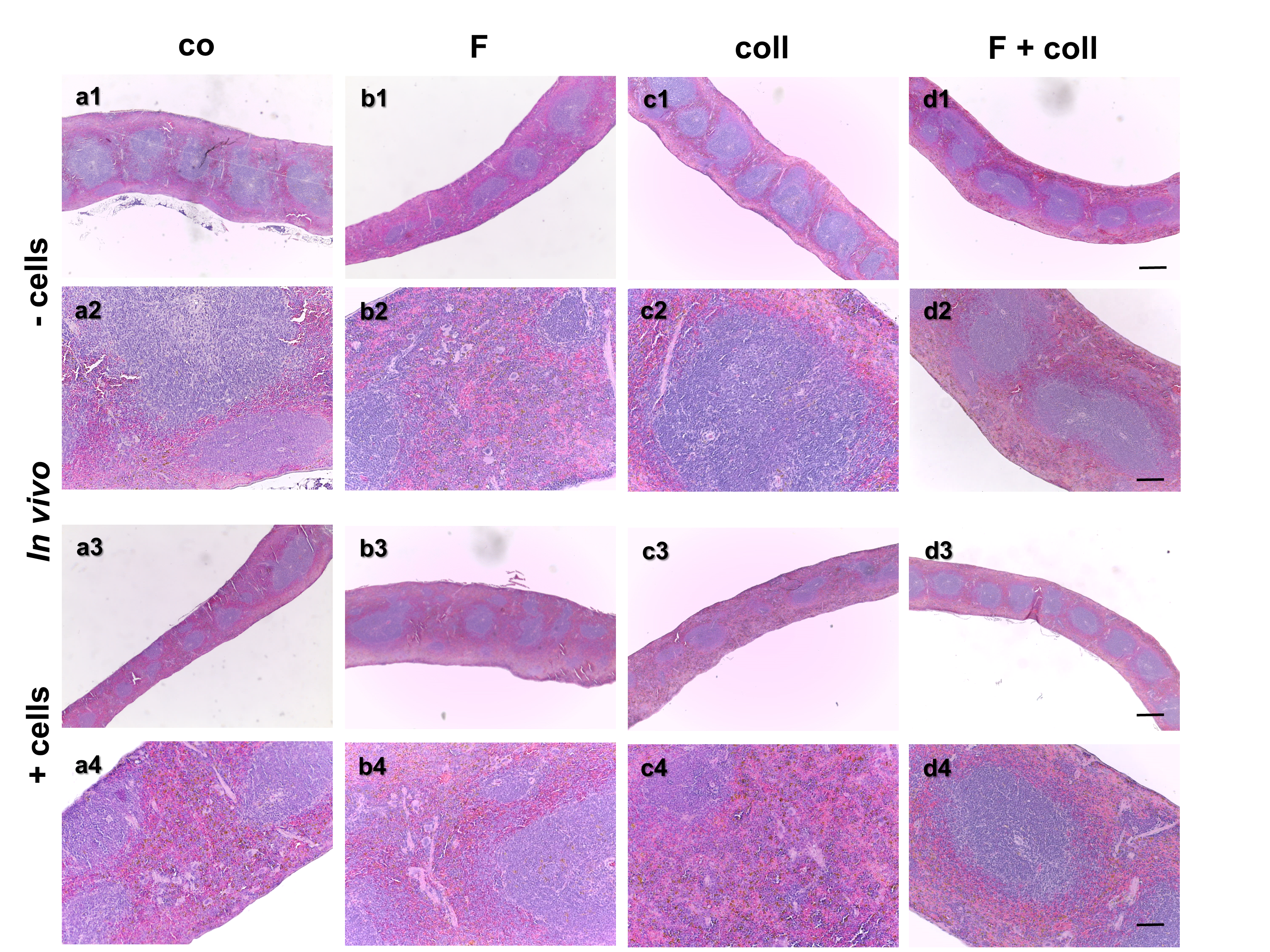

Supplement: Supplementary file 4 — Supplementary file4 Supplemental figure 4: Histopathology of the spleen of mice 3 months after implantation of the different scaffold variants, depicted by HE staining. Scaffolds without cells (-cells, a1–d2) and seeded with LACL-derived ligamentocytes, cultured for one week on the scaffold before scaffold implantation (+cells, a3–d4). a: co, controls; b: F, functionalization by gas-phase fluorination; c: coll, collagen foam cross-linked with HMDI; d: collagen foam combined with gas-phase fluorination. Scale bars 100 µm (a1–d1, a3–d3), 50 µm (a2–d2, a4–d4). (TIF 9844 KB) [file 418_2022_2156_MOESM4_ESM.tif]

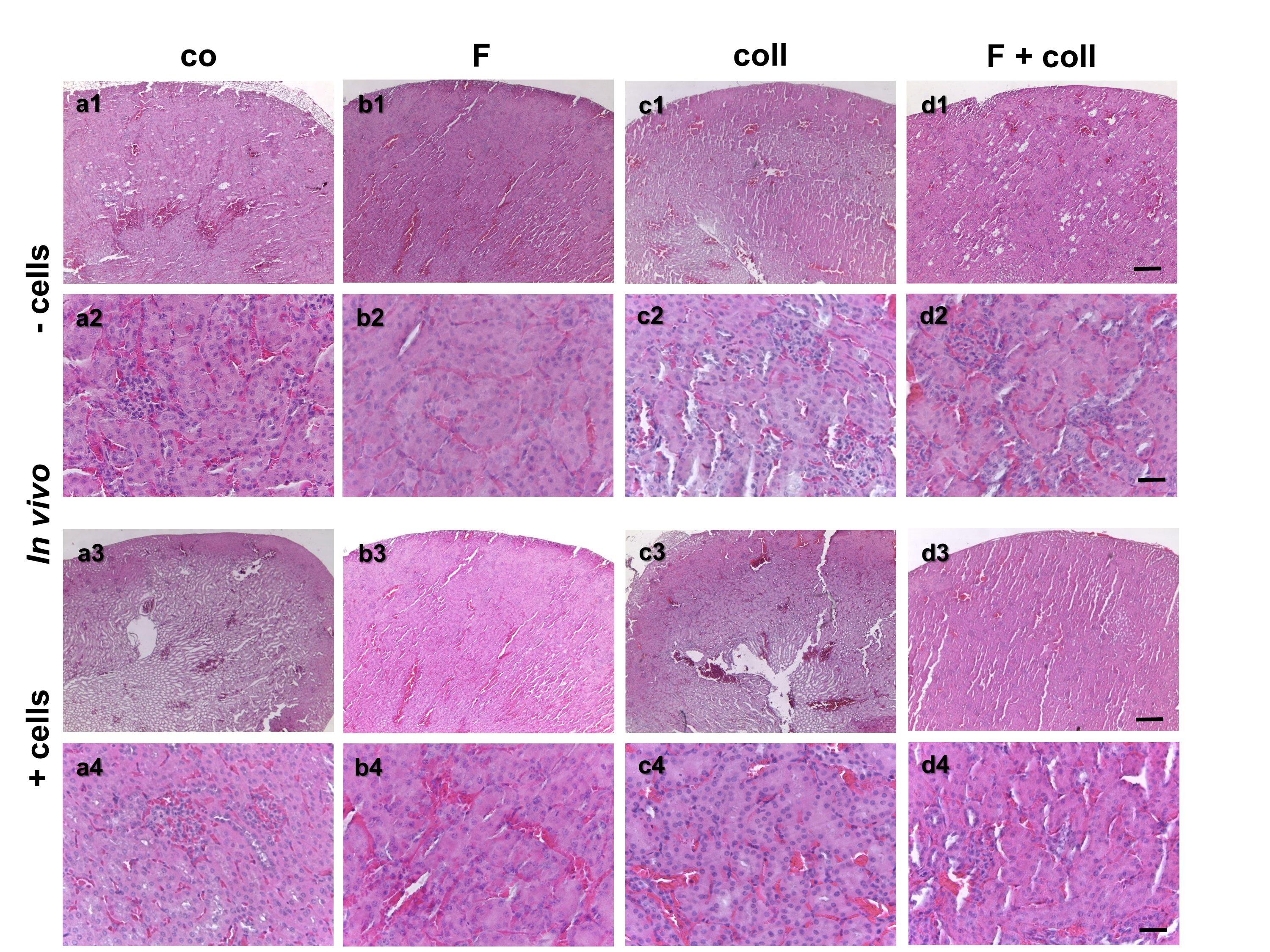

Supplement: Supplementary file 5 — Supplementary file5 Supplemental figure 5: Histopathology of the kidney of mice 3 month after implantation of the different scaffold variants, depicted by HE staining. Scaffolds without cells (-cells, a1–d2) and seeded with LACL-derived ligamentocytes, cultured for one week on the scaffold before scaffold implantation (+cells, a3–d4). a: co, controls; b: F, functionalization by gas-phase fluorination; c: coll, collagen foam cross-linked with HMDI; d: collagen foam combined with gas-phase fluorination. Scale bars: 100 µm (a1–d1, a3–d3), 50 µm (a2–d2, a4–d4: the renal cortex is shown at larger magnification). (TIF 11542 KB) [file 418_2022_2156_MOESM5_ESM.tif]

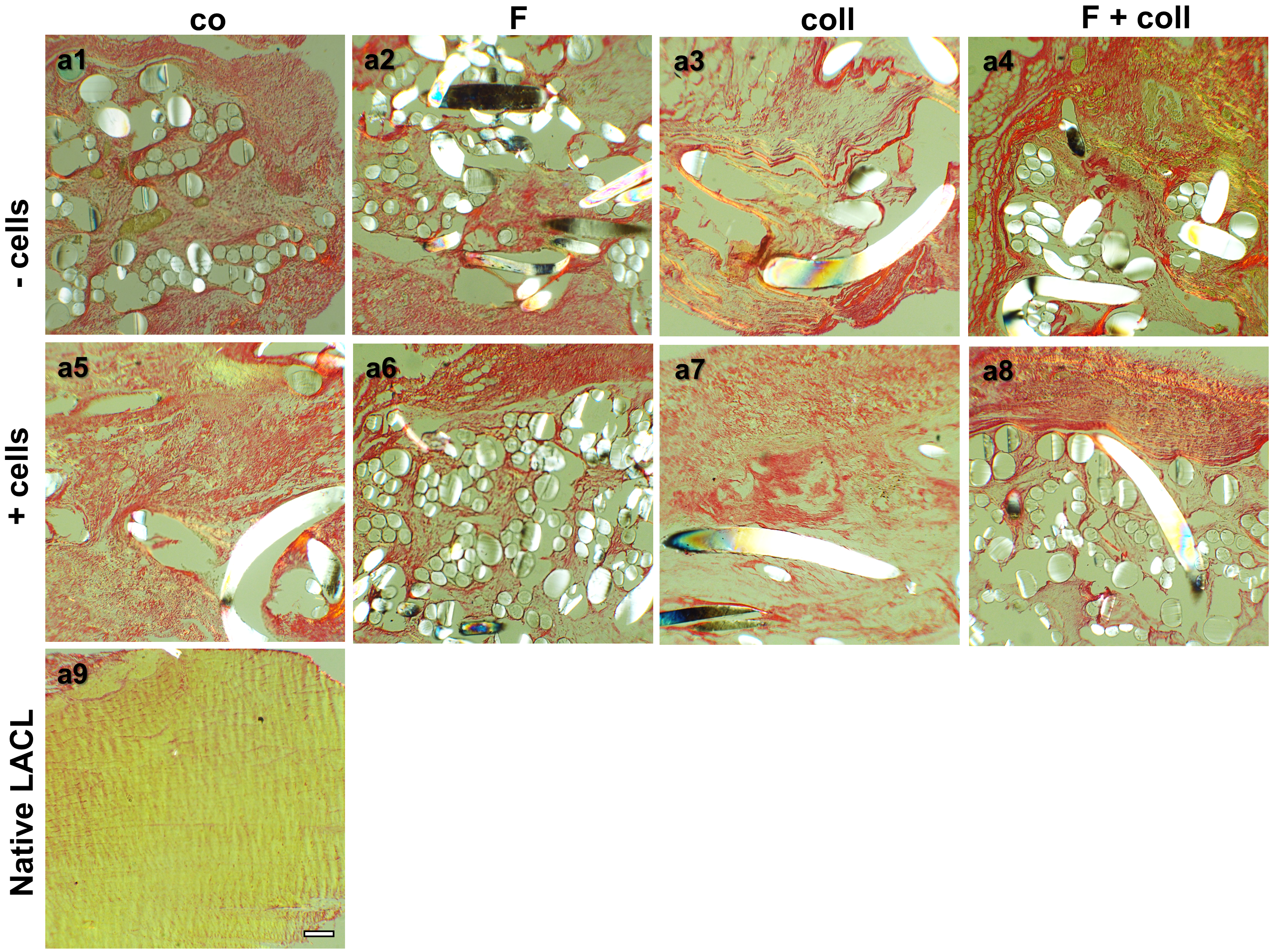

Supplement: Supplementary file 6 — Supplementary file6 Supplemental Figure 6: Visualization of collagen by polarization microscopy in the different scaffold variants explanted after 3 months in vivo and stained with Sirius red (SR). SR stain to visualize collagen organization of the tissue within the scaffold variants without cells (a1–a4) and implanted with lapine anterior cruciate ligament (LACL)-derived ligamentocytes, cultured for one week on the scaffold in vitro before explanted after 12 weeks in vivo (a5–a8), co (a1, a5), F (a2, a6), coll (a3, a7) and F + coll (a4, a8). Native LACL (a9). Scale bars 100 µm (a1–a9). (TIF 11087 KB) [file 418_2022_2156_MOESM6_ESM.tif]
